# Impaired β-cell glucokinase as an underlying mechanism in diet-induced diabetes

**DOI:** 10.1242/dmm.033316

**Published:** 2018-06-13

**Authors:** Brian Lu, Kiran Kurmi, Miguel Munoz-Gomez, Egon J. Jacobus Ambuludi, Jason M. Tonne, Kuntol Rakshit, Taro Hitosugi, Yogish C. Kudva, Aleksey V. Matveyenko, Yasuhiro Ikeda

**Affiliations:** 1 Department of Molecular Medicine, Mayo Clinic, Rochester, MN 55905, USA; 2 Virology and Gene Therapy Graduate Program, Mayo Clinic Graduate School of Biomedical Sciences, Rochester, MN 55905, USA; 3 Molecular Pharmacology and Experimental Therapeutics Graduate Program, Mayo Clinic Graduate School of Biomedical Sciences, Rochester, MN 55905, USA; 4 Department of Physiology and Biomedical Engineering, Mayo Clinic, Rochester, MN 55905, USA; 5 Department of Molecular Pharmacology and Experimental Therapeutics, Mayo Clinic, Rochester, MN 55905, USA; 6 Division of Endocrinology, Diabetes, Metabolism, and Nutrition, Mayo Clinic, Rochester, MN 55905, USA

**Keywords:** Glucokinase, Diet-induced diabetes, Insulin secretion, Islet biology, Gene therapy

## Abstract

High-fat diet (HFD)-fed mouse models have been widely used to study early type 2 diabetes. Decreased β-cell glucokinase (GCK) expression has been observed in HFD-induced diabetes. However, owing to its crucial roles in glucose metabolism in the liver and in islet β-cells, the contribution of decreased GCK expression to the development of HFD-induced diabetes is unclear. Here, we employed a β-cell-targeted gene transfer vector and determined the impact of β-cell-specific increase in GCK expression on β-cell function and glucose handling *in vitro* and *in vivo*. Overexpression of GCK enhanced glycolytic flux, ATP-sensitive potassium channel activation and membrane depolarization, and increased proliferation in Min6 cells. β-cell-targeted GCK transduction did not change glucose handling in chow-fed C57BL/6 mice. Although adult mice fed a HFD showed reduced islet GCK expression, impaired glucose tolerance and decreased glucose-stimulated insulin secretion (GSIS), β-cell-targeted GCK transduction improved glucose tolerance and restored GSIS. Islet perifusion experiments verified restored GSIS in isolated HFD islets by GCK transduction. Thus, our data identify impaired β-cell GCK expression as an underlying mechanism for dysregulated β-cell function and glycemic control in HFD-induced diabetes. Our data also imply an etiological role of GCK in diet-induced diabetes.

This article has an associated First Person interview with the first author of the paper.

## Abstract (precis)

**Summary:** β-cell glucokinase expression is decreased in diet-induced diabetes. β-cell-targeted overexpression of glucokinase improved the diabetic phenotype, suggesting an etiological role of glucokinase downregulation in diet-induced diabetes.

## INTRODUCTION

The link between glucose metabolism and high dietary fat is well established in animal models (Lichtensteina and Schwabb, 2000). High-fat diet (HFD) feeding in mice leads to obesity and insulin resistance, and is commonly used as a model for diet-induced diabetes (Peyot et al., 2010; Surwit et al., 1988; Winzell and Ahrén, 2004). β-cells in mice initially respond to the increased insulin demand with compensatory β-cell proliferation (Ahrén et al., 2010), but with chronic HFD, failing β-cell function eventually leads to glucose intolerance and hyperglycemia (Peyot et al., 2010; Winzell and Ahrén, 2004). This HFD-induced defect in β-cell function has previously been linked to downregulation of islet glucokinase (GCK), a key regulator of glucose metabolism. HFD feeding reduces *Gck* mRNA and GCK protein by up to 45% compared with isoenergetic high-carbohydrate feeding in rats (Kim et al., 1995). *Ex vivo* islet studies also show reduction in *Gck* mRNA and GCK protein after co-culture with palmitate (Gremlich et al., 1997; Yoshikawa et al., 2001). In accordance with these observations, reduced GCK expression in islets has been found in patients with type 2 diabetes (Del Guerra et al., 2005; Gunton et al., 2005; Taneera et al., 2014). However, owing to the expression of GCK in both β-cells and hepatocytes, elucidating the contributions of altered GCK expression/activity to HFD-induced diabetes in a β-cell-specific manner has been challenging. In particular, the etiological role of impaired GCK expression in HFD-induced diabetes remains poorly understood.

In this study, we employed a β-cell-targeted adeno-associated viral (AAV) vector system (Tonne et al., 2015) and determined the impact of increased β-cell-specific GCK expression on β-cell function in HFD-induced diabetes. Our results demonstrate that improved GCK expression in β-cells restores glucose-stimulated insulin secretion (GSIS), lowers fasting blood glucose and improves glucose tolerance in a mouse model of HFD-induced diabetes, indicating a crucial role of impaired β-cell GCK expression in diet-induced diabetes.

## RESULTS

### GCK increases glycolytic flux, intracellular Ca^2+^ concentration and β-cell proliferation

**Fig. 1.**
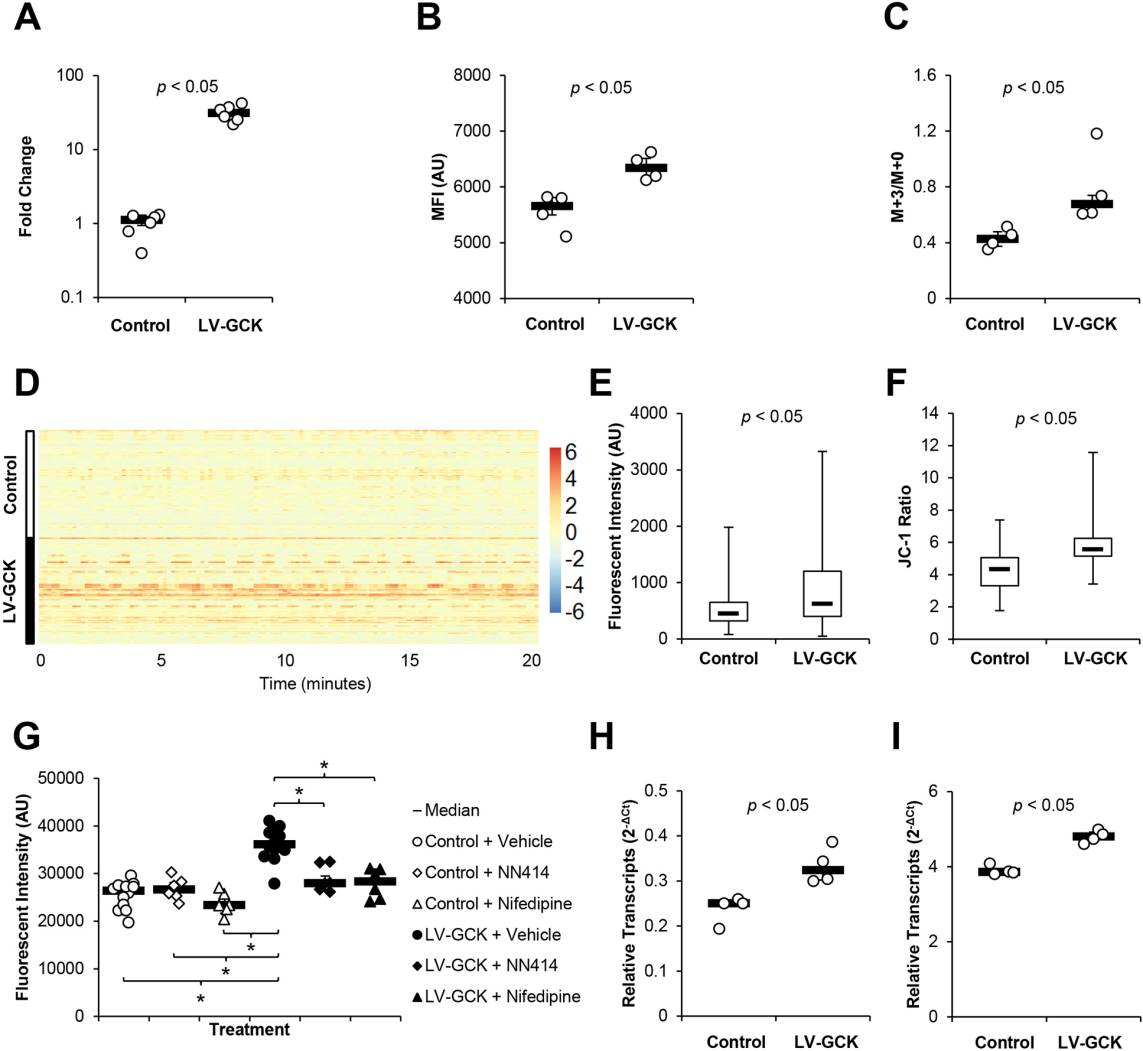
**GCK increases glycolytic flux and enhances β-cell proliferation.** GCK was overexpressed in Min6 cells by transduction with lentiviral vector expressing GCK under the mouse insulin 2 promoter (SIN-mIP2-GCK). (A) Densitometry analysis of immunoblot of nontransduced control Min6 cells and Min6 cells transduced with lentiviral vector SIN-mIP2-GCK. (B) Mean fluorescent intensity (MFI; in arbitrary units, AU) of Min6 cells as measured by flow cytometry following overnight incubation with fluorescent glucose analog 2-deoxy-2-[(7-nitro-2,1,3-benzoxadiazol-4-yl)amino]-D-glucose (2-NBDG) (*n*=4 per group). (C) Ratio of ^13^C-labeled lactate to unlabeled lactate following overnight incubation with U-13C6 D-glucose was measured by gas chromatography mass spectrometry (GC/MS) (*n*=4 per group). (D) Single-cell Ca^2+^-dependent RCaMP1h fluorescent intensity trace (*n*=210-213 per group). Color scale indicates z-scores normalized per time frame. (E) Fluorescent image analysis of median Ca^2+^-dependent RCaMP1h fluorescent intensity during the trace shown in D. (F) JC-1 ratio of nontransduced control Min6 cells and Min6 cells overexpressing GCK was determined using a JC-1 Mitochondrial Membrane Potential Assay Kit (Cayman Chemical). (G) Relative cell numbers following incubation with vehicle, NN414 or nifedipine was determined using the CyQUANT NF Proliferation Assay (Thermo Fisher Scientific) 2 days after seeding (*n*=6-12 per group). (H) Relative expression of *Irs2* transcripts in nontransduced control and GCK-overexpressing Min6 cells (*n*=4 per group). (I) Relative expression of *Ccnd2* transcripts in nontransduced control and GCK-overexpressing Min6 cells (*n*=4 per group). Results are shown as scatter plots with medians (black bars)±MAD, heatmap or box plots. Whiskers represent minimum and maximum values. *P<0.05.

To understand the effect of GCK overexpression in β-cells, we first increased GCK expression in a β-cell line *in vitro*. Min6 cells were transduced with lentiviral vector expressing GCK (LV-GCK) and selected using puromycin. Transduction by GCK-expressing lentiviral vector resulted in enhanced GCK expression in Min6 cells (Fig. 1A; Fig. S1). When incubated with the fluorescent glucose analog 2-deoxy-2-[(7-nitro-2,1,3-benzoxadiazol-4-yl)amino]-D-glucose (2-NBDG), GCK overexpression significantly enhanced the fluorescent intensity of Min6 cells, indicating increased glucose uptake by GCK overexpression (Fig. 1B). Because GCK catalyzes the first step of glycolysis, we then tested the glycolytic flux of GCK-overexpressing Min6 cells by ^13^C-glucose tracing. After incubating the cells with U-13C6 D-glucose for 18 h, we found significantly increased ^13^C-labeled lactate (Fig. 1C), indicating increased glycolytic flux in GCK-overexpressing Min6 cells. To determine relative changes in intracellular Ca^2+^ concentration, we transduced Min6 cells with lentiviral vectors expressing the Ca^2+^-dependent fluorescent protein RCaMP1h and selected using puromycin, establishing the Min6 RCaMP1h cell line. Similar expression of RCaMP1h in control Min6 RCaMP1h cells and Min6 RCaMP1h cells further transduced with lentiviral vector expressing GCK was verified using quantitative reverse transcription polymerase chain reaction (RT-qPCR) (Fig. S2A,B). Analysis of live-cell images of Min6 RCaMP1h cells and Min6 RCaMP1h cells overexpressing GCK showed no change in glucose-dependent Ca^2+^ flux (Fig. S3A,B), which corresponded with the lack of changes in GSIS found in these cells (Fig. S3C). However, we observed increased Ca^2+^-dependent fluorescence in RCaMP1h-expressing Min6 cells overexpressing GCK (Fig. 1D,E; Figs S2C and S3B), indicating elevated cytoplasmic Ca^2+^ concentration. Min6 cells overexpressing GCK also showed an increased JC-1 ratio, indicating an increase in mitochondrial potential-dependent JC-1 aggregation (Fig. 1F). Vector-mediated GCK overexpression also led to an increase in relative cell counts 48 h after cell seeding (Fig. 1G). Inhibition of ATP-sensitive potassium (K_ATP_) or Ca^2+^ channel depolarization with 200 µM NN414 or 28.9 µM nifedipine, respectively, abolished the increase in relative cell count in GCK-overexpressing Min6 cells without increasing cytotoxicity (Fig. 1G; Fig. S4), suggesting roles of K_ATP_ or Ca^2+^ channel depolarization in GCK-mediated enhancement of β-cell proliferation. RT-qPCR of GCK-overexpressing Min6 cells showed elevated levels of *Irs2* and *Ccnd2* transcripts (Fig. 1H,I), which is consistent with previous studies implicating the cyclin D2 pathway in glucose-mediated β-cell proliferation (Porat et al., 2011; Salpeter et al., 2011; Stamateris et al., 2016).

### β-cell-targeted GCK transduction has minimal effects on β-cell function in chow-fed mice

**Fig. 2.**
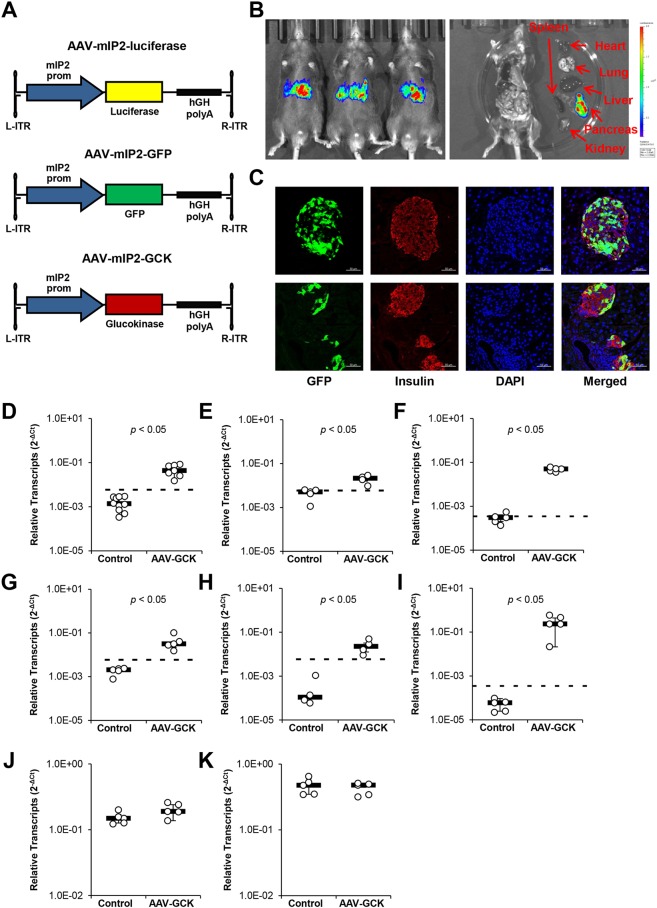
**mIP2-restricted AAV expression.** PBS control or AAV vectors were administered via intraperitoneal injection. Mice were sacrificed for analysis 2 weeks after injection. (A) Schematics of AAV vector constructs with mIP2 driving expression of luciferase (AAV-mIP2-luciferase, top), GFP (AAV-mIP2-GFP, middle), and mouse GCK (AAV-mIP2-GCK, bottom) followed by the human growth hormone (hGH) poly-A. (B) Luminescence imaging of whole mice injected with AAV-mIP2-luciferase. Color scale represents luminescence in radiance (p/s/cm^3^/sr; minimum, 2×10^5^; maximum, 2×10^6^). (C) Representative fluorescent confocal microscopy images of pancreatic islets (top) with surrounding exocrine tissue (bottom) from AAV-mIP2-GFP-treated mice. Green, GFP; red, insulin; blue, DAPI. Scale bars: 50 µm. (D) Relative expression of AAV-specific *Gck* transcripts (GenBank BC011139.1) in pancreases of 8-month-old mice fed a chow diet (*n*=7-10 per group). (E) Relative expression of AAV-specific *Gck* transcripts (GenBank BC011139.1) in isolated islets of 8-month-old mice fed a chow diet (*n*=4-5 per group). (F) Relative expression of AAV-derived *Gck* transcripts in livers of 8-month-old mice fed a chow diet (*n*=5 per group). (G) Relative expression of AAV-specific *Gck* transcripts (GenBank BC011139.1) in pancreases of 8-month-old mice fed a HFD (*n*=5 per group). (H) Relative expression of AAV-specific *Gck* transcripts (GenBank# BC011139.1) in isolated islets of 8-month-old mice fed a HFD (*n*=5 per group). (I) Relative expression of AAV-derived *Gck* transcripts in livers of 8-month-old mice fed a HFD (*n*=5 per group). (J) Relative expression of total (endogenous and exogenous) *Gck* transcripts in livers of 8-month-old mice fed a chow diet (*n*=5 per group). (K) Relative total expression of total (endogenous and exogenous) *Gck* transcripts in livers of 8-month-old mice fed a HFD (*n*=5 per group). Dashed lines indicate the limit of detection. Results are shown as scatter plots with medians (black bars)±MAD.

**Fig. 3.**
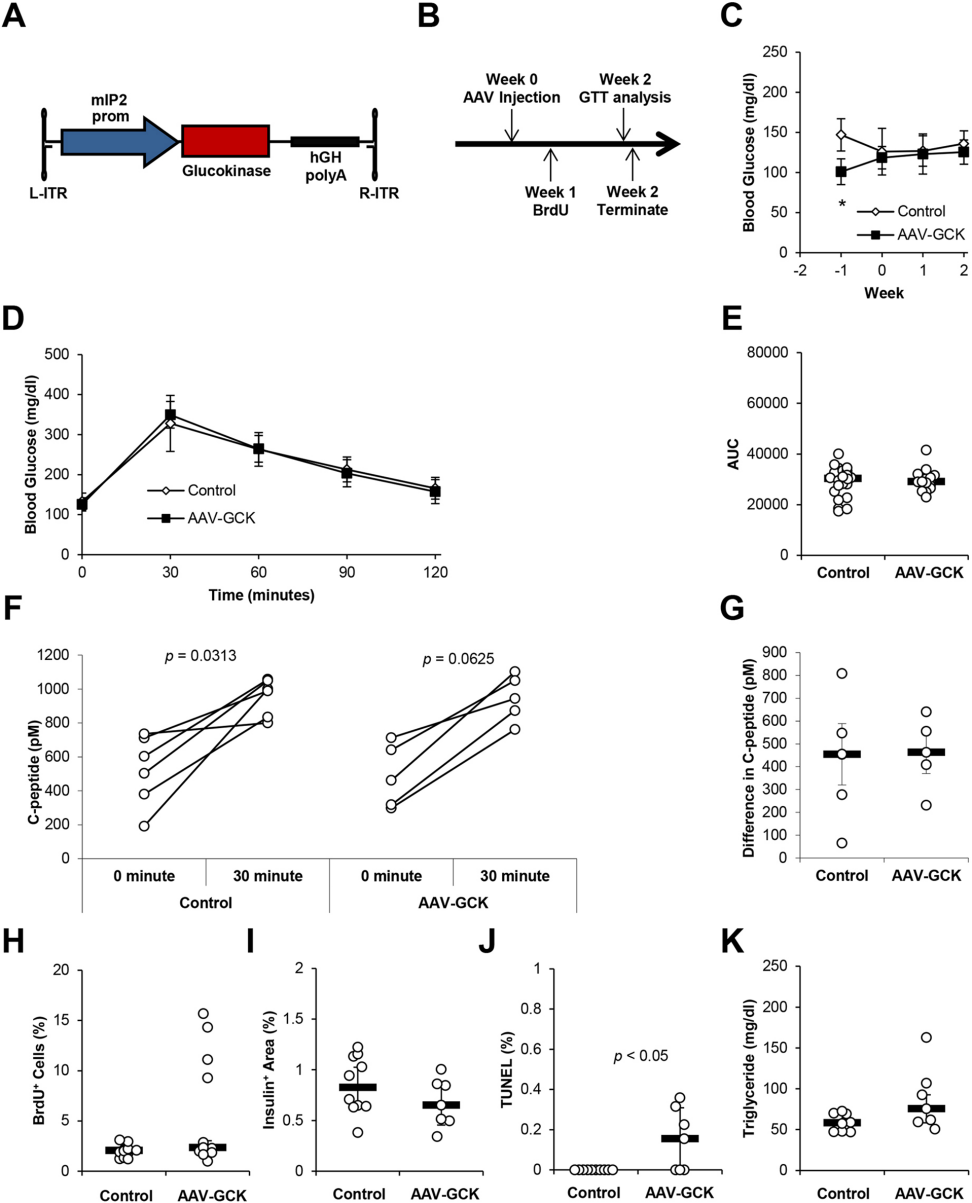
**GCK overexpression has minimal effects on β-cell proliferation and function in mice fed a chow diet.** (A) Schematic of AAV-mIP2-GCK vector construct with mIP2 driving expression of mouse GCK followed by the hGH poly-A. (B) Mice were administered PBS control or 3.3×10^13^ AAV-mIP2-GCK genome copy (gc)/kg body weight via intraperitoneal injection. BrdU was administered through drinking water *ad libitum* 1 week after AAV injection. Intraperitoneal glucose tolerance test (IPGTT) was performed 2 weeks following AAV injection. Mice were then killed for analysis. (C) Weekly blood glucose was measured following a 6-h fast. (D) IPGTT after a 6-h fast 2 weeks following AAV delivery (*n*=16-25 per group). (E) Area-under-the-curve analysis of the IPGTT shown in D (*n*=16-25 per group). (F) Plasma C-peptide before and 30 min after injection of glucose during IPGTT (*n*=5-6 per group). (G) Difference between plasma C-peptide before and 30 min after injection of glucose during IPGTT (*n*=5-6 per group). (H) Insulin^+^ BrdU^+^ cells as a percentage of insulin^+^ cells was counted from fluorescent confocal microscopy images of mouse pancreas sections (*n*=11-13 per group). (I) Insulin^+^ area as a percentage of total pancreas area was determined by anti-insulin HRP staining of mouse pancreas sections (*n*=7-10 per group). (J) Percentage of TUNEL^+^ insulin^+^ cells was counted from fluorescent confocal microscopy images of mouse pancreas sections (*n*=7-10 per group). (K) Plasma triglyceride concentration was measured 2 weeks after vector injection (*n*=7-8 per group). Results are shown as scatter plots with medians (black bars)±MAD or line graphs with medians±MAD, *P<0.05.

We next tested the effects of GCK overexpression in β-cells of chow-fed mice. We employed the β-cell-targeted adeno-associated viral (AAV) vector system, which facilitated selective transgene expression under the control of the mouse *Ins2* promoter (mIP2) (El Khatib et al., 2015; Tonne et al., 2015). mIP2 promoter restriction was confirmed by luciferase imaging of mice treated with AAV serotype 8 (AAV8) vectors expressing luciferase and GFP, AAV-mIP2-luciferase and AAV-mIP2-GFP (Fig. 2A-C). We generated β-cell-targeted AAV8 vector expressing mouse GCK, AAV-mIP2-GCK, which was then delivered via intraperitoneal injection (Fig. 3A,B). Mice were killed for analysis after the vector was allowed to express for 2 weeks. Efficient AAV vector transduction of the pancreas and increased *Gck* expression were confirmed by RT-qPCR (Fig. 2D-E; Fig. S5). RT-qPCR also confirmed no changes in liver *Gck* expression following AAV vector delivery (Fig. 2J), although a low level of AAV-derived *Gck* transcripts was detectable (Fig. 2F). β-cell-targeted GCK transduction did not cause hypoglycemia, and intraperitoneal glucose tolerance test (IPGTT) conducted 2 weeks post vector administration showed no changes in glucose tolerance (Fig. 3C-E). Similarly, no change was detected in plasma C-peptide during IPGTT (Fig. 3F,G). To assess β-cell proliferation, BrdU was introduced in the drinking water 1 week after vector administration for 1 week. Confocal microscopy analysis showed no significant difference in the percentage of insulin^+^ cells that were BrdU^+^ (Fig. 3H; Fig. S6). We also found no changes in insulin^+^ area (Fig. 3I; Fig. S7). Together with the increase in insulin^+^ TUNEL^+^ apoptotic cells (Fig. 3J; Fig. S8), we concluded that β-cell-targeted GCK transduction leads to an increase in β-cell turnover, without strongly affecting glucose metabolism in chow-fed mice. To check for hypertriglyceridemia, we also measured triglyceride concentrations in plasma. No changes in plasma triglyceride concentrations were detected (Fig. 3K).

### Increased GCK expression in HFD-induced diabetes restores β-cell function and improves glucose tolerance

**Fig. 4.**
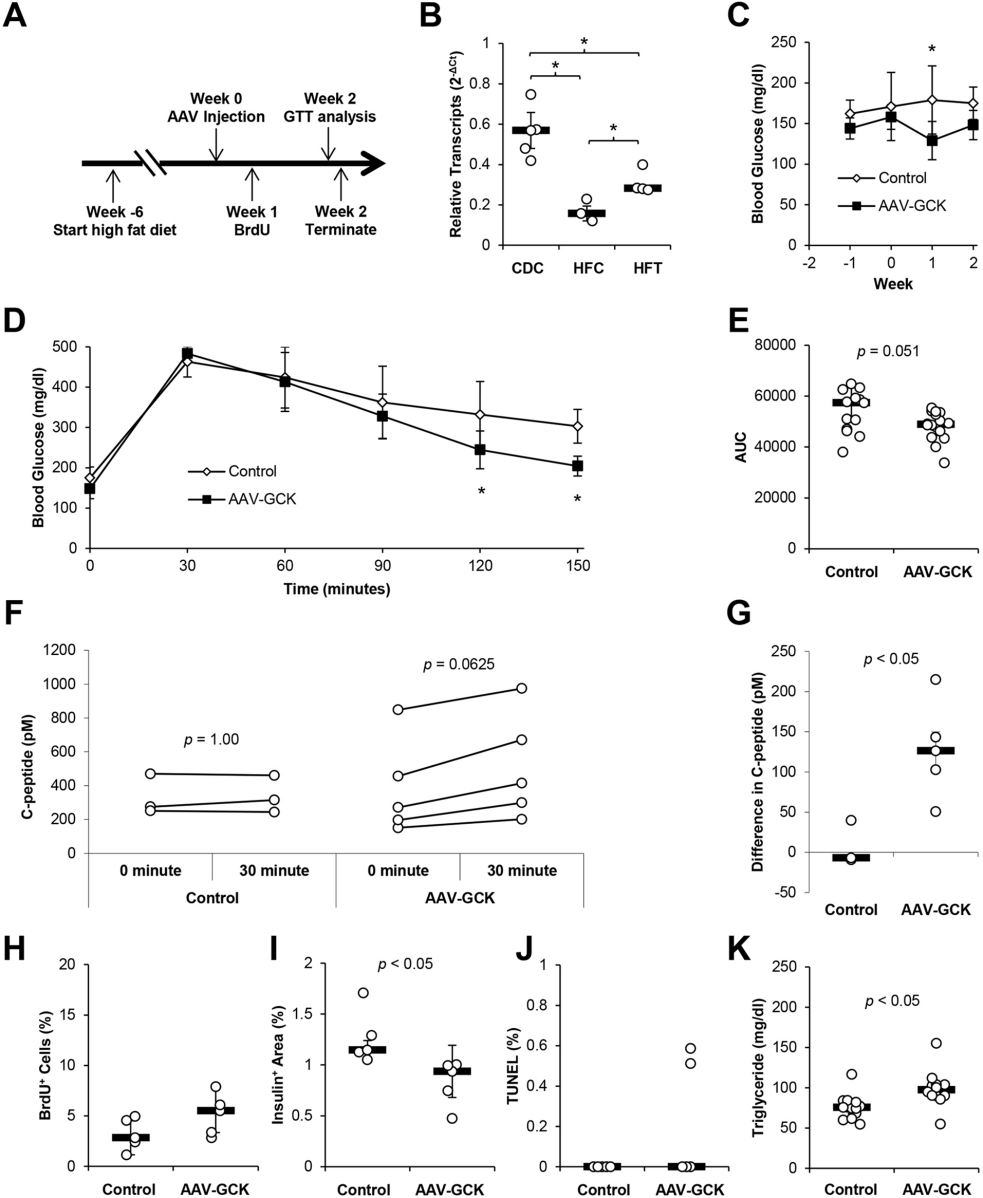
**Increased GCK expression in HFD-induced diabetes restores β-cell function and improves glucose tolerance.** (A) HFD was fed *ad libitum* for at least 6 weeks prior to AAV injection. Mice were given PBS control or 3.3×10^13^ AAV-mIP2-GCK genome copy (gc)/kg body weight via intraperitoneal injections. BrdU was administered through drinking water *ad libitum* 1 week after AAV injection. IPGTT was performed 2 weeks following AAV injection. Mice were then killed for analysis. (B) Relative expression of *Gck* transcripts in isolated islets 2 weeks after AAV injection. CDC, chow diet controls; HFC, HFD controls; HFT, HFD treated. (C) Weekly blood glucose was measured following a 6-h fast. (D) IPGTT after a 6-h fast 2 weeks following AAV delivery (*n*=16-18 per group). (E) Area-under-the-curve analysis of IPGTT shown in D (*n*=16-18 per group). (F) Plasma C-peptide before and 30 min after injection of glucose during IPGTT (*n*=3-5 per group). (G) Difference between circulating C-peptide before and 30 min after injection of glucose during IPGTT (*n*=3-5 per group). (H) Insulin^+^ BrdU^+^ cells as a percentage of insulin^+^ cells was counted from fluorescent confocal microscopy images of mouse pancreas sections (*n*=5 per group). (I) Insulin^+^ area as a percentage of total pancreas area was determined by anti-insulin HRP staining of mouse pancreas sections (*n*=5 per group). (J) Percentage of TUNEL^+^ insulin^+^ cells was counted from fluorescent confocal microscopy images of mouse pancreas sections (*n*=5 per group). (K) Plasma triglyceride concentration was measured 2 weeks after vector injection (*n*=12-13 per group). Results are shown as scatter plots with medians (black bars)±MAD or line graphs with medians±MAD, *P<0.05.

We then determined the role of GCK expression in HFD-induced diabetes. Mice were fed a HFD for 6 weeks, followed by AAV-mIP2-GCK vector or mock treatment for 2 weeks (Fig. 4A). Mice fed a HFD for 6 weeks had increased body weight, elevated fasting glucose and impaired IPGTT compared with age-matched mice fed a chow diet (Fig. S9). Efficient AAV vector transduction of the pancreas and increased *Gck* expression in HFD mice were confirmed by RT-qPCR (Fig. 2G-H; Fig. S5). RT-qPCR verified reduced islet *Gck* expression in HFD-fed mice compared with chow-fed mice and increased *Gck* expression 2 weeks following AAV transduction (Fig. 4B). Similar to observations in chow-diet mice, no changes in liver *Gck* were detected following AAV vector injection, despite a small, but detectable, level of AAV-derived *Gck* transcripts in the liver (Fig. 2I,K). β-cell-targeted GCK transduction significantly reduced fasting blood glucose levels 1 week after vector administration (Fig. 4C). When IPGTT was conducted 2 weeks post vector administration, blood glucose levels 120 min and 150 min after glucose injection were also significantly lower than those of HFD controls (Fig. 4D), resulting in a trend towards decreased area under the curve (Fig. 4E). In comparison, AAV-mediated expression of ANGPTL8 (betatrophin), which does not affect β-cell function (Cox et al., 2016; Gusarova et al., 2014; Liu et al., 2018), had no effect on glucose tolerance test in HFD mice in parallel experiments, suggesting that the improved glucose clearance was not caused by transduction by AAV (Fig. S10). These observations indicate improved glucose clearance following GCK overexpression. Concomitantly, GCK-overexpressing HFD mice showed an increased median difference in plasma C-peptide 30 min following glucose injection, suggesting improved GSIS in vector-treated HFD mice (Fig. 4F,G). Although HFD increased the insulin^+^ area in control mice compared with chow-fed control mice as expected (Fig. S9E), AAV-mIP-2-GCK administration led to a small but significant decrease in insulin^+^ area in HFD mice without changing insulin^+^ BrdU^+^, and insulin^+^ TUNEL^+^, staining (Fig. 4H-J; Fig. S6-S8). Notably, a significant increase was detected in plasma triglycerides (control, 75.6 mg/dl; treated, 97.4 mg/dl) (Fig. 4K). The improved GSIS despite decreased insulin^+^ area suggests that enhanced β-cell GCK expression improves glucose handling in HFD-induced diabetes through improved β-cell function rather than increased β-cell mass.

### β-cell-targeted GCK expression re-establishes the GSIS capacity of HFD mouse islets *ex vivo*

**Fig. 5.**
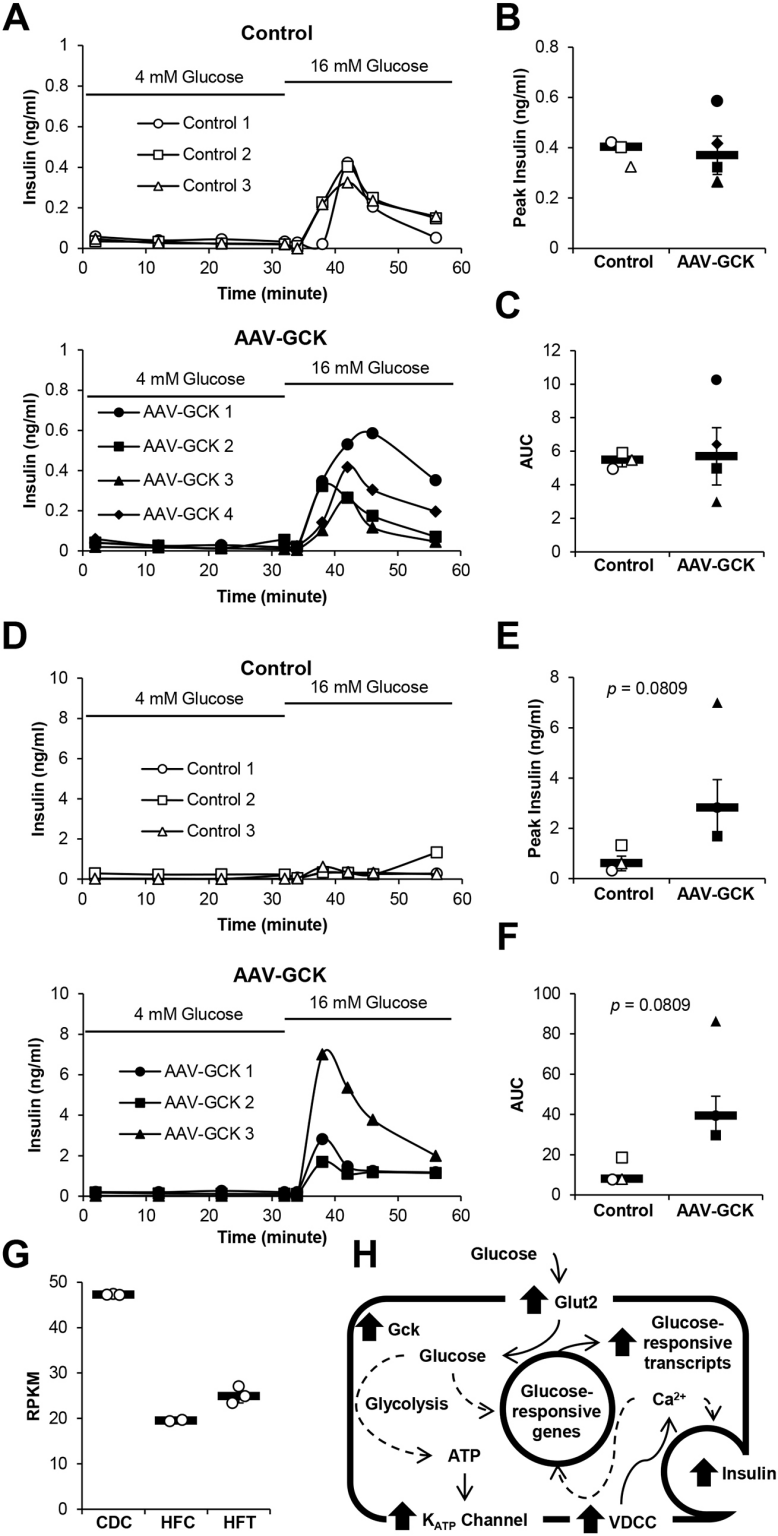
**β-cell-targeted GCK expression re-established the GSIS capacity of HFD mouse islets *ex vivo*.** (A) Insulin secretion during islet perifusion of islets from PBS control (top) and AAV-treated (bottom) mice fed a chow diet 2 weeks following AAV delivery in 4 mM glucose medium followed by 16 mM glucose medium (*n*=3-4 per group). (B) Peak insulin secretion during perifusion with 16 mM glucose medium shown in A (*n*=3-4 per group). (C) Area-under-the-curve analysis of insulin secretion shown in A (*n*=3-4 per group). (D) Insulin secretion during islet perifusion of islets from PBS control (top) and AAV-treated (bottom) mice fed a HFD 2 weeks following AAV delivery in 4 mM glucose medium followed by 16 mM glucose medium (*n*=3 per group). (E) Peak insulin secretion during perifusion with 16 mM glucose medium shown in D (*n*=3 per group). (F) Area-under-the-curve analysis of insulin secretion shown in D (*n*=3 per group). (G) RNA sequencing was performed using RNA isolated from islets of chow diet control mice (CDC), HFD control mice (HFC) and HFD AAV-mIP2-GCK-treated mice (HFT) 2 weeks following AAV delivery. *Gck* RPKM determined by RNA sequencing analysis. (H) Summary of the pathway analysis of islet transcriptome upon GCK transduction. Major changes in cellular pathways affected by GCK expression are shown. Dashed and solid line arrows indicate indirect and direct interactions, respectively. Bolded arrows indicate upregulation of the indicated pathways. Results are shown as line graphs representing individual mouse or scatter plots with medians (black bar)±MAD.

To determine whether β-cell-targeted GCK transduction restores islet GSIS, islets were purified from mice fed chow diet or HFD and subjected to islet perifusion. Islets from chow-fed mice showed typical patterns of GSIS upon perifusion with 16 mM glucose (Fig. 5A). Corresponding with the *in vivo* GSIS results (Fig. 3G), AAV-mIP2-GCK vector treatment did not enhance insulin secretion during 16 mM glucose stimulation (Fig. 5B,C). Perifusion with 16 mM glucose medium showed little insulin secretion from control HFD mouse islets (Fig. 5D-F). In sharp contrast, islets from AAV-mIP2-GCK-treated mice fed a HFD showed high levels of insulin secretion upon stimulation with 16 mM glucose medium (Fig. 5D-F). Our data verified that β-cell-targeted GCK transduction improves β-cell GSIS in HFD-induced diabetes.

### Insulin secretion and insulin signaling pathway genes are upregulated by β-cell-targeted GCK expression in HFD-induced diabetes

To determine the mechanism underlying improved GSIS with GCK expression, we performed RNA sequencing analysis on isolated mouse islets. Corresponding with our RT-qPCR results (Fig. 4B), we detected a decrease in median *Gck* reads per kilobase of transcript per million mapped reads (RPKM) in HFD control islets (HFC) (42% of chow) compared with chow diet control islets (CDC), which was partially restored (53% of chow) after vector-mediated GCK expression (Fig. 5G). Pathway analysis was conducted using generally applicable gene set enrichment for pathway analysis (GAGE), with pathways derived from the Kyoto Encyclopedia of Genes and Genomes (KEGG) database (Kanehisaa and Goto, 2000; Luo et al., 2009). Nine KEGG pathways, including insulin secretion, insulin signaling and cAMP pathways, were upregulated and 39 KEGG pathways were downregulated following GCK expression in HFD islets (Table 1; Fig. S4). HFD islet expression profiles of 94 genes previously associated with islet dysfunction also partially normalized to those of chow diet control islets with AAV-mediated GCK expression (Fig. S11) (Taneera et al., 2012). Further transcriptome analysis demonstrated clear trends in increased transcripts of K_ATP_ and voltage-dependent Ca^2+^ channel genes, glucose-responsive genes and Ca^2+^/calmodulin-dependent protein kinase pathway genes, in addition to enhanced glucose metabolism, insulin secretory and insulin signaling pathways (Figs S12 and S13).

**Table 1.**
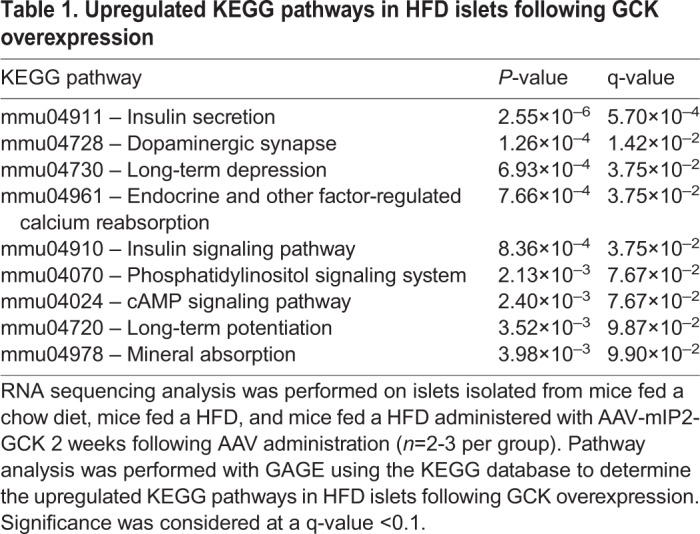
**Upregulated KEGG pathways in HFD islets following GCK overexpression**

## DISCUSSION

The importance of GCK in glucose metabolism is exemplified by the inverse relationship between fasting glucose and GCK gene copy number in transgenic mouse models (Niswender et al., 1997a). Heterozygous GCK knockout impairs β-cell function and results in diabetes (Bali et al., 1995; Terauchi et al., 1995), while homozygous GCK knockout is lethal owing to severe diabetes (Postic et al., 1999). Conversely, increased GCK gene copy number results in over 25% reduction in fasting blood glucose levels and a fourfold increase in hepatic glycogen synthesis with no change in circulating C-peptide levels (Niswender et al., 1997b). This indicates that increased GCK gene dosage reduces blood glucose levels primarily through increased hepatic glucose metabolism rather than enhanced β-cell function (Niswender et al., 1997b). Our study showed no notable changes in glucose handling or GSIS, as well as no hypoglycemic episodes, in chow-fed mice following β-cell-targeted GCK overexpression. These observations further support the notion that enhanced hepatic GCK, but not β-cell GCK, induces hypoglycemia in normoglycemic animals. Our study also revealed that impaired GCK expression in β-cells plays a crucial role in diet-induced diabetes in this mouse model.

Reduced islet GCK expression has been found in patients with type 2 diabetes (Del Guerra et al., 2005; Gunton et al., 2005; Taneera et al., 2014). Our data and previous reports show reduced β-cell GCK expression by HFD feeding (Gremlich et al., 1997; Kim et al., 1995). Reduced islet GCK expression is also linked to impaired GSIS (Terauchi et al., 2007; Yoshikawa et al., 2001). Our finding of restored β-cell function by β-cell-targeted GCK overexpression suggests an etiological role of GCK suppression in β-cell dysfunction in HFD-induced diabetes. This mechanism is likely regulated through PDX-1, which controls GCK expression (Watada et al., 1996) and is downregulated in diabetes (Taneera et al., 2014). Incubation of rat islets in fatty acids also decreases PDX-1 expression, leading to reduced GCK expression (Gremlich et al., 1997). It is conceivable that restoring GCK expression increases glycolytic flux and induces glucose-responsive genes (Schuit et al., 2002; Webb et al., 2000), such as PDX-1, leading to restoration of β-cell function. Interestingly, improved GSIS only led to a modest improvement in GTT in our HFD-fed mice. However, this is likely to have been caused by our use of IPGTT instead of oral glucose tolerance test (OGTT). OGTT, unlike IPGTT, induces an incretin response and potentiates stronger GSIS (Andrikopoulos et al., 2008). It is likely that the use of OGTT in GCK-overexpressing HFD mice would result in higher GSIS, and potentially better improved GTT, than was observed in our IPGTT owing to the induction of incretins.

We also showed that β-cell-specific GCK overexpression can enhance β-cell function depending on diet conditions. While GCK overexpression had minimal effects on glucose metabolism in mice fed a chow diet, GCK overexpression enhanced β-cell function in HFD mice. The determining factor for whether GCK overexpression enhances β-cell proliferation or β-cell function is likely the resulting changes in glycolytic flux. Glucose is well known to regulate β-cell proliferation and GSIS (Matschinsky et al., 1993; Porat et al., 2011; Salpeter et al., 2011; Stamateris et al., 2016), and we show that modulating GCK expression can change β-cell glycolytic flux (Becker et al., 1996; Wang and Iynedjian, 1997). By increasing β-cell GCK expression above normal physiological levels found in chow-diet β-cells, β-cell glucose utilization is increased, and glucose induces β-cell proliferation through induction of IRS2 and cyclin D2 (Matschinsky et al., 1993; Porat et al., 2011; Salpeter et al., 2011; Stamateris et al., 2016; Terauchi et al., 2007). However, in our HFD β-cells, GCK expression was already below normal physiological levels. By increasing GCK expression towards normal levels, we were able to restore β-cell function, likely by increasing β-cell glucose utilization.

The increase in TUNEL staining in mice fed a chow diet could be a sign of increased glycolytic flux due to overexpressing GCK above physiological levels, as hyperglycolysis has been shown to result in DNA damage, leading to β-cell death via p53 activation (Tornovsky-Babeay et al., 2014). However, the increased TUNEL staining appears to be sufficiently counterbalanced by β-cell proliferation, as mice fed a chow diet did not show any impairment in glucose tolerance. This is similar to the case of an activating GCK mutation identified in a patient with neonatal hypoglycemia treated with subtotal pancreatectomy (Kassem et al., 2010). The activating mutation resulted in enlarged islets owing to enhanced β-cell proliferation despite increased β-cell apoptosis. Interestingly, we observed a small, but significant, difference between the β-cell areas of HFD control and treated mice despite no change in TUNEL staining. Additionally, both cell proliferation pathways (cell cycle and DNA replication) and cell death pathways (p53 signaling and apoptosis) in HFD islets were downregulated following vector treatment. This, in conjunction with lower fasting blood glucose and improved glucose tolerance, suggests that GCK overexpression reduced the HFD-stimulated compensatory increase in β-cell area, similar to what was previously proposed for a glucokinase activator (GKA) (Nakamura et al., 2009). Surprisingly, glycolysis and oxidative phosphorylation pathways were also downregulated in treated HFD islets. However, these are suggestive of normalization of β-cell pathways rather than β-cell dysfunction, as glycolysis and oxidative phosphorylation pathways were both upregulated in islets following a HFD (Figs S14 and S15). Notably, the minor hexokinase isozymes in β-cells, the expression of which can shift the glucose dose response curve for GSIS out of physiological range (Becker et al., 1996; Muller et al., 2014), were upregulated following a HFD but downregulated following treatment. Upregulation of oxidative phosphorylation following a HFD, which can lead to glucotoxicity via oxidative stress, resulting in impaired GSIS (Robertson, 2004), was similarly downregulated following treatment.

Studies with GKAs in animals and humans have demonstrated reduced blood glucose levels, enhanced β-cell function and proliferation. Several clinical GKA trials have experienced declining efficacy, hypoglycemia and increased plasma triglycerides after prolonged treatment (Agius, 2014). Because hepatic GCK overexpression increases hepatic lipogenesis and plasma levels of triglycerides in rats (O'Doherty et al., 1999), increased hepatic triglyceride production, or steatosis by liver-specific GCK, has been proposed as the likely cause for elevated plasma triglycerides and declining efficiency (Agius, 2014; Meininger et al., 2011). Our data demonstrate that β-cell-targeted GCK overexpression shows beneficial effects in HFD-induced diabetes with improved β-cell function upon GCK transduction. However, although β-cell-targeted GCK transduction did not induce hypoglycemia, elevated plasma triglyceride concentrations were observed in treated animals. While AAV-derived *Gck* transcripts were detectable in the livers of our treated mice via RT-qPCR, the level of AAV-mediated *Gck* expression was not sufficient to influence total (endogenous and exogenous) liver *Gck* expression (Fig. 2J,K), suggesting that the level of AAV-mediated *Gck* expression in the liver was negligible. As such, the increase in plasma triglycerides is likely not caused by direct modulation of hepatic GCK activity. Insulin has been shown to enhance hepatic release of triglycerides (Topping and Mayes, 1972). β-cell-targeted GCK transduction might indirectly induce hepatic GCK activity through insulin from improved β-cell function, and, similar to hepatic GCK overexpression, the increase in hepatic GCK activity could then lead to increased hepatic glycolytic flux, resulting in increased plasma triglyceride (O'Doherty et al., 1999). Our data therefore suggest that β-cell-specific GCK activation or β-cell-targeted GCK gene therapy might still be complicated by hypertriglyceridemia.

In summary, our data further highlight the effects of β-cell GCK expression on β-cell function. Improved β-cell GCK expression restored GSIS and improved glucose tolerance in HFD-induced diabetes, suggesting an etiological role of GCK suppression in β-cell dysfunction in HFD-induced diabetes. Further understanding of the mechanism of hypertriglyceridemia induction upon β-cell-targeted GCK expression could lead to therapeutic modulation of β-cell GCK for diet-induced diabetes.

## MATERIALS AND METHODS

### Animals

All animal experiments were approved by the Mayo Clinic Institutional Animal Care and Use Committee. Six-month-old male C57BL/6j mice were purchased from the Jackson Laboratory and further aged to at least 8 months prior to vector administration. Mice were maintained on a PicoLab Rodent Diet 20 (LabDiet) or OpenSource Diets D12331 58% fat diet (Research Diets). D12331 diet was maintained for at least 6 weeks prior to the start of experiments.

### Cells

293T cells were purchased from American Type Culture Collection and cultured in Dulbecco's modified Eagle medium (DMEM) supplemented with 10% fetal bovine serum (FBS), 50 U/ml penicillin and 50 µg/ml streptomycin. Min6 cells (a gift from Dr Raymond Hickey, Mayo Clinic, Rochester, MN, USA) were cultured in DMEM supplemented with 10% heat-inactivated FBS, 50 U/ml penicillin, 50 µg/ml streptomycin, and 50 pM beta-mercaptoethanol. Islets were cultured in RPMI supplemented with 10% FBS, 50 U/ml penicillin and 50 µg/ml streptomycin.

### Plasmids

To generate GCK-expressing lentiviral vector, the mouse GCK encoding sequence (GenBank BC011139.1) was amplified by PCR and cloned into pHRSINCSGW_PGRPuro (Sakuma et al., 2014). The mIP2 sequence was amplified by fusion PCR to disrupt the internal EcoRI site and cloned into the GCK open reading frame encoding lentiviral vector construct, resulting in pSIN-mIP2-GCK. The plasmid construct pRSET-RCaMP1h was a kind gift from Dr Loren Looger through Addgene (#42874) (Akerboom et al., 2013). RCaMP1h was amplified by PCR and cloned into pHRSIN-CSGW_PGRPuro (Sakuma et al., 2014), resulting in pSIN-SFFV-RcaMP1h. GCK-expressing AAV vector construct was produced by cloning PCR-amplified mouse GCK sequence (GenBank BC011139.1) into pAAV-mIP2-Luc, resulting in pAAV-mIP2-GCK (Tonne et al., 2015). PCR primer sequences used for cloning are listed in Table S1. Codon-optimized ANGPTL8 was synthesized by GenScript and cloned into the BamHI-XhoI sites of pAAV-CMV-eGFP (Schreiber et al., 2015), resulting in pAAV-CMV-ANGPTL8.

### Lentiviral and AAV vector production

Lentiviral vectors were produced by three-plasmid transfection as previously described with minor modifications (Tonne et al., 2011). Briefly, vector plasmid pSIN-mIP2-GCK or pSIN-SFFV-RCaMP1h and packaging plasmids Ex-QV and pMD-G were co-transfected into 293T cells. Lentiviral vectors were purified by filtration and concentrated by ultracentrifugation. AAV8 vectors were produced by three-plasmid transfection as previously described with minor modifications (Cataliotti et al., 2011). Briefly, vector plasmid pAAV-mIP2-GCK or pAAV-CMV-ANGPTL8, AAV8 capsid plasmid pRep2Cap8 (kindly provided by Dr Michael A. Barry, Mayo Clinic, Rochester, MN, USA), and helper plasmid pHelper (Stratagene) were co-transfected into 293T cells. Cell-associated AAV vectors were concentrated via ultracentrifugation, and desalted by filtration.

### *In vitro* studies with Min6 cells

GCK-overexpressing Min6 cells and calcium-reporter Min6 RCaMP1h cells were generated by transducing Min6 cells with the lentiviral vectors expressing mouse GCK or RCamP1h, respectively, followed by puromycin selection. GCK overexpression was confirmed via immunoblotting. Immunoblots were imaged with a biostep CELVIN S Chemiluminescence Imager using biostep SnapAndGo software (ver. 1.6.2 rev. 10). Densitometry analyses were performed using ImageJ (ver. 1.51u) and normalized to Ponceau S total protein staining. To measure cell proliferation, 5000 nontransduced control or GCK-overexpressing Min6 cells per well were seeded onto 96-well plates. Vehicle, NN414 or nifedipine were added 6 h after seeding at the indicated final concentrations. Cell proliferation was measured by CyQUANT NF Proliferation Assay (Thermo Fisher Scientific) 2 days after cell seeding. To measure potential cytotoxicity associated with ion channel inhibitors, cells were seeded and incubated with vehicle, NN414 or nifedipine as described above. Cytotoxicity was then measured using CytoTox-Glo Cytotoxicity Assays (Promega) 2 days after cell seeding. Glucose uptake was determined by flow cytometry analysis of cells after overnight culture with 121.7 µM 2-NBDG (Cayman Chemical). ^13^C-glucose tracing in Min6 cells was quantified with gas chromatography mass spectrometry as previously described (Hitosugi et al., 2012), following overnight culture with 24.1 mM U-13C6 D-glucose (Cambridge Isotope Laboratories). The glycolysis flux rate was determined by dividing the ion intensity of [^3-13^C]-lactate (m/z 222) by that of [^12^C]-lactate (m/z 219). Natural isotope abundance was calculated with IsoPat^2^ software using pure lactate (Gruber et al., 2007). To assess mitochondrial membrane potential, 5×10^4^ nontransduced control Min6 cells or GCK-overexpressing Min6 cells per well were seeded onto 96-well plates and allowed to adhere overnight. Mitochondrial membrane potential was measured the following morning using a JC-1 Mitochondrial Membrane Potential Assay Kit (Cayman Chemical). RNA transcript expression was quantified by SYBR green-based RT-qPCR as previously described, with minor modifications (Tonne et al., 2015). Briefly, RNA was extracted using RNeasy Plus Mini kits (Qiagen). cDNA was synthesized using the RNA to cDNA EcoDry Premix (Oligo dT) kit (Clontech). Primers used for RT-qPCR are listed in Table S3.

### Live-cell fluorescent microscopy

Live-cell imaging of Min6 cells expressing the RCamP1h fluorescent calcium reporter (Min6 RCaMP1h cells) was performed using the Nikon Biostation IM-Q. Min6 RCaMP1h control cells or Min6 RCaMP1h cells overexpressing GCK (1×10^6^ to 2×10^6^) were seeded onto an ibidi µ-Dish and allowed to adhere overnight. Min6 RCaMP1h cells were then imaged at the indicated glucose concentration. Min6 RCaMP1h cells were imaged every 10 s for the indicated amount of time. Fluorescent intensities of RCaMP1h were then measured from individual cells using the NIS Elements BR software (ver. 3.22.14 build 736, Nikon) at each time interval. Heatmaps of z-scores of the fluorescent intensity of individual cells over time were generated using the Bioconductor package ‘pheatmap’ (https://CRAN.R-project.org/package=pheatmap).

### *In vivo* studies

Mice were administered 3.3×10^13^ AAV-mIP2-GCK genome copy (gc) per kg body weight via intraperitoneal injection. PBS control and AAV treatment groups were randomly assigned. Then, 0.2% (g/v) 2-bromo-2′deoxyuridine (BrdU) supplemented with 1% (g/v) glucose was administered in the drinking water 1 week after vector administration. IPGTTs were performed as previously described 2 weeks after vector delivery (Tonne et al., 2015). Blood glucose was monitored using a FreeStyle Lite Blood Glucose Monitor (Abbott Laboratories). GSIS was measured by C-peptide enzyme-linked immunosorbent assay (ELISA) (ALPCO) using plasma isolated from whole blood collected at 0 min and 30 min. Plasma triglyceride concentrations were measured by a Triglyceride Colorimetric Assay Kit (Cayman Chemical) using plasma isolated from whole blood collected 2 weeks after AAV administration.

### β-cell proliferation, apoptosis and insulin^+^ area measurement

Mouse pancreases were mounted, sectioned and immunostained as previously described, with minor modifications (Tonne et al., 2015). Antibodies used for immunostaining are listed in Table S2. Briefly, 7 µm pancreas sections were fixed in 4% paraformaldehyde, permeabilized in 0.5% Triton X-100 and blocked with 5% FBS in PBS. Insulin^+^ cells were stained by Dako guinea pig anti-insulin antibody (1:400; Agilent Technologies). BrdU^+^ β-cells were stained by rat anti-BrdU antibody (1:100; Abcam), visualized by fluorescent confocal microscopy and counted manually. Proliferating β-cells were counted as insulin^+^ cells co-localized with BrdU and 4′,6-diamidino-2-phenylindole (DAPI) staining. Insulin^+^ area was visualized via horseradish peroxidase (HRP) staining using a DAB Peroxidase (HRP) Substrate Kit (Vector Laboratories). HRP slides were then imaged using an Aperio ScanScope AT Turbo Scanner (Leica Biosystems). β-cell area was measured using ImageJ (ver. 1.51u) as the percentage of insulin^+^ area as visualized by HRP staining in the entire pancreas section. β-cell apoptosis was determined using a TMR Red *In Situ* Cell Death Detection Kit (Roche). Apoptotic β-cells were counted as insulin^+^ cells co-localized with Tdt-mediated dUTP-X nick end labeling (TUNEL) (TMR Red *In Situ* Cell Death Detection Kit, Roche) and DAPI staining.

### *Ex vivo* perifusion assay

Islet perifusion assays were performed as previously described (Lu et al., 2018). Briefly, mouse islets were isolated from C57BL/6j mice and cultured overnight in RPMI supplemented with 10% FBS, 50 U/ml penicillin and 50 µg/ml streptomycin. The following morning, 20 size-matched islets were picked for islet perifusion experiments. Islets were washed in 4 mM glucose medium for 48 min. Samples were then collected every 2 min while islets were exposed to 4 mM glucose medium for 32 min, followed by 16 mM glucose medium for 32 min. Insulin secretion at select time points was determined by mouse insulin ELISA (ALPCO).

### Viral genome and expression quantitation from mouse pancreas

Genomic DNA was extracted from pancreas sections with the PureLink Genomic DNA Mini Kit (Thermo Fisher Scientific). AAV genome copies were quantified using TaqMan quantitative PCR as previously described (Tonne et al., 2013). Primers used for AAV genome copy quantification are listed in Table S3. RNA was extracted from pancreas sections and expression quantified by RT-qPCR as previously described with minor modifications (Tonne et al., 2015). Briefly, RNA was extracted from pancreas sections using RNeasy Plus Mini Kits (Qiagen). cDNA was synthesized using the RNA to cDNA EcoDry Premix (Oligo dT) kit (Clontech) or the SuperScript III Reverse Transcriptase (Invitrogen). Transcript expression was quantified using SYBR green-based RT-qPCR. Primers used for RT-qPCR are listed in Table S3.

### RNA sequencing and analysis

RNA was extracted from leftover islets from islet perifusion experiments using an RNeasy Plus Mini Kit (Qiagen). cDNA library construction and next generation sequencing was conducted by the Mayo Clinic Medical Genome Facility Gene Expression and Sequencing Cores. cDNA library was prepared using the TruSeq RNA Library Prep Kit v2 (Illumina) following analysis of RNA quality with an Agilent 2100 Bioanalyzer and sequenced with HiSeq 4000 (Illumina). Raw sequencing data were aligned by the Mayo Clinic Bioinformatics Core using TopHat (Trapnell et al., 2012). The aligned genes were analyzed with EdgeR (Robinson et al., 2010). Pathway analysis was conducted with GAGE (Luo et al., 2009). KEGG pathway graphs were generated using Pathview (Kanehisaa and Goto, 2000; Luo and Brouwer, 2013), and heatmaps were generated using the Bioconductor package ‘pheatmap’. Raw and normalized data were deposited in Gene Expression Omnibus (GEO), under accession number GSE104581.

### Data analysis

Data are presented as scatterplots with median±median absolute deviation (MAD), line graphs with median±MAD, or box plots with whiskers showing minimums and maximums. Scatterplots were drawn according to Weissgerber et al. (2015). Data were analyzed using the JMP software (ver. 13.0.0) or the *R* statistical language (R Development Core Team, 2017). As not all data were normally distributed as determined using Shapiro–Wilk tests, statistical significance was analyzed using the nonparametric two-tailed Wilcoxon rank-sum test or two-tailed Wilcoxon signed-rank test as appropriate. Multiple comparisons were analyzed by Kruskal–Wallis test followed by Conover-Iman test with Holm-Šidák correction. *P*<0.05 was considered significant.

## Supplementary Material

Supplementary information

First Person interview
