# Supplementary material for: Impaired β-cell glucokinase as an underlying mechanism in diet-induced diabetes
Source: Dis Model Mech. 2018 Jun 13;11(6):dmm033316. doi: 10.1242/dmm.033316 (PMC6031355; doi:10.1242/dmm.033316)
Supplement: Supplementary information [file dmm-11-033316-s1.pdf]

Table S1. PCR primers.

Product	Vector	Cloning Sites	Primer Pair
Mouse *Gck*	Lentiviral	BamHI-NotI	Forward 5'-TCAGGGATCCACCATGGCTGTGGATA -3'<br>Reverse 5'-GATTGCGGCCGCTCACTGGCCCAGC -3'
Mouse insulin 2 gene promoter	Lentiviral	EcoRI-BamHI	Forward 5'-CGCGAATTCCCCTCCTCTTGCATTTCA-3'<br>Reverse 5'-AGGAAAGCAGAATTTAGGCAGCAAGGCACT-3'<br>Forward 5'-AGTGCCTTGCTGCCTAAATTCTGCTTTCCT-3'<br>Reverse: 5'-TCCACAGGATCCTGTTGAAACAATAA-3'
RCaMP1h	Lentiviral	BamHI-NotI	Forward 5'-CAACAGGATCCACCATGGGTTCTCATCATCATCAT-3',<br>Reverse 5'-GAGTCGCGGCCGCTTACTTCGCTGTCATCATTTG-3'
Mouse *Gck*	AAV	BamHI-XhoI	Forward 5'-TCAGGGATCCACCATGGCTGTGGATA-3'<br>Reverse 5'-GATTCTCGAGTCACTGGCCCAGCATG-'3

PCR amplification primer pairs used for cloning into lentiviral or AAV vectors. The mouse insulin 2 gene promoter was amplified by fusion PCR to disrupt the internal EcoRI site (5'-GAATTC-3' to 5'-AAATTC-3') prior to cloning into the EcoRI-BamHI sites in the GCK ORF encoding lentiviral vector construct.

Table S2. Antibodies.

Antibody	Company	Application	Product Number	Dilution
Mouse anti-GCK IgG	R&D Systems	Immunoblot	MAB7840	1:500
Goat anti-mouse HRP IgG	Jackson ImmunoResearch	Immunoblot	115-035-174	1:5000
Guinea pig anti-insulin pAb	Dako	Immunostaining	A056401	1:400
Rat anti-BrdU IgG	Abcam	Immunostaining	ab6326	1:100
Donkey anti-rat AF 594 IgG	Invitrogen	Immunostaining	A-21209	1:1000
Donkey anti-guinea pig AF 488 IgG	Jackson ImmunoResearch	Immunostaining	706-546-148	1:1000
Donkey anti-guinea pig HRP IgG	Jackson ImmunoResearch	Immunostaining	706-035-148	1:1000

Antibodies used for immunoblot and immunostaining. Antibodies for immunoblot were diluted in PBS supplemented with 5% Blotting Grade Blocker (Bio-Rad). Antibodies for immunostaining were diluted in PBS supplemented with 5% FBS.

Disease Models & Mechanisms • Supplementary information

Table S3. qPCR and RT-qPCR primers.

Gene	Vendor	Sequence/Product Number
Mouse *Gck*	IDT	Mm.PT.58.33016327
Mouse *Irs2*	IDT	Mm.PT.58.31740094
Mouse *Ccnd2*	IDT	Mm.PT.58.10811551
Mouse *Hprt*	IDT	Mm.PT.39a.22214828
AAV genome	IDT	Forward: 5'-CCTGGGTTCAAGCGATTCTC-3'<br>Reverse: 5'-AGCTGAGCCTGGTCATGCAT-3'<br>TaqMan Probe: TGCCTCAGCCTCCCGAGTTGT
AAV-specific *Gck* (GenBank# BC011139.1)	IDT	Forward: 5'-GGCTGTGGATACTACAAGG-3'<br>Reverse: 5'-ATCACCTTCTTCAGGTCTTC-3'
AAV-derived *Gck*	IDT	Forward: 5'-CAAGAAGGCTTGCATGCTG-3'<br>Reverse: 5'-TTCCAGGGCCAGGAGAG-3'
AAV-derived *Angptl8*	IDT	Forward: 5'-GAACTGACTCTGCTCTTCCAC-3'<br>Reverse: 5'-CTGTACCGAGAAACTCCAGTG-3'
Mouse *Angptl8*	QIAGEN	QT01533777

Primer pairs used for qPCR and RT-qPCR. DNA was extracted from pancreas sections using PureLink Genomic DNA Mini Kits (Thermo Fisher Scientific) and quantified using TaqMan qPCR. RNA was extracted from cells, pancreas sections, liver sections, and mouse islets using RNeasy Plus Mini Kits (QIAGEN). cDNA was synthesized using the RNA to cDNA EcoDry Premix (Oligo dT) kit (Clontech) or the SuperScript III Reverse Transcriptase (Invitrogen). Transcript expression was quantified using SYBR green-based RT-qPCR or TaqMan-based qPCR. AAV-specific *Gck* primers binds exons 1-2 of *Gck* (GenBank# BC011139.1). AAV-derived *Gck* primers bind exon 10 of *Gck* (GenBank# BC011139.1) and the poly(A) tail sequence in the AAV construct.

Disease Models & Mechanisms • Supplementary information

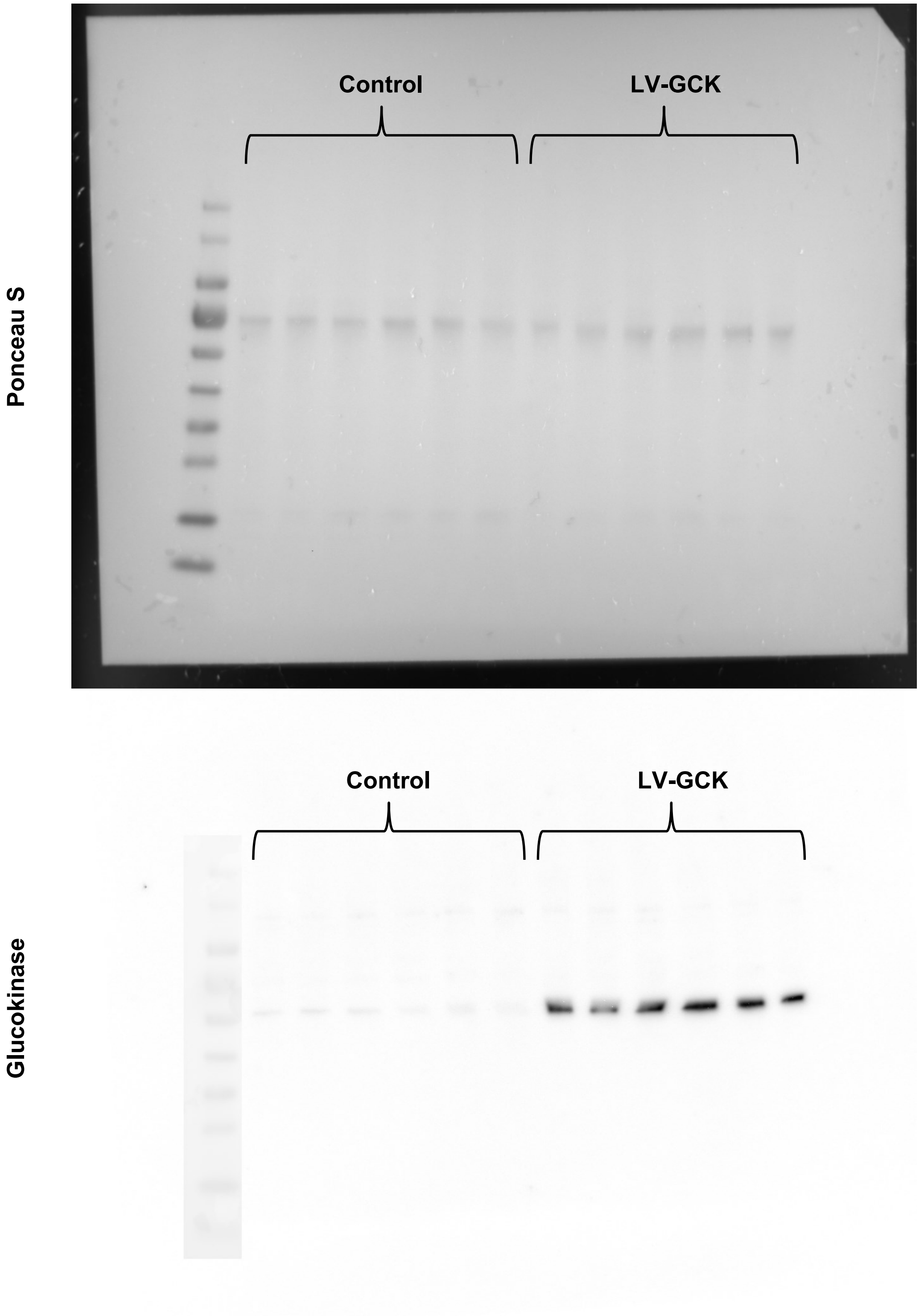

Figure S1. Raw total protein staining and immunoblot images for densitometry analysis. Total protein was visualized using Ponceau S (top) (Thermo Fisher Scientific). Blots were imaged using a biostep CELVIN S Chemiluminescence Imager and analyzed in ImageJ (*n* = 6 per group). Image of the protein ladder was superimposed onto the immunoblot image (bottom) using the biostep SnapAndGo control software (ver. 1.6.2 rev. 10).

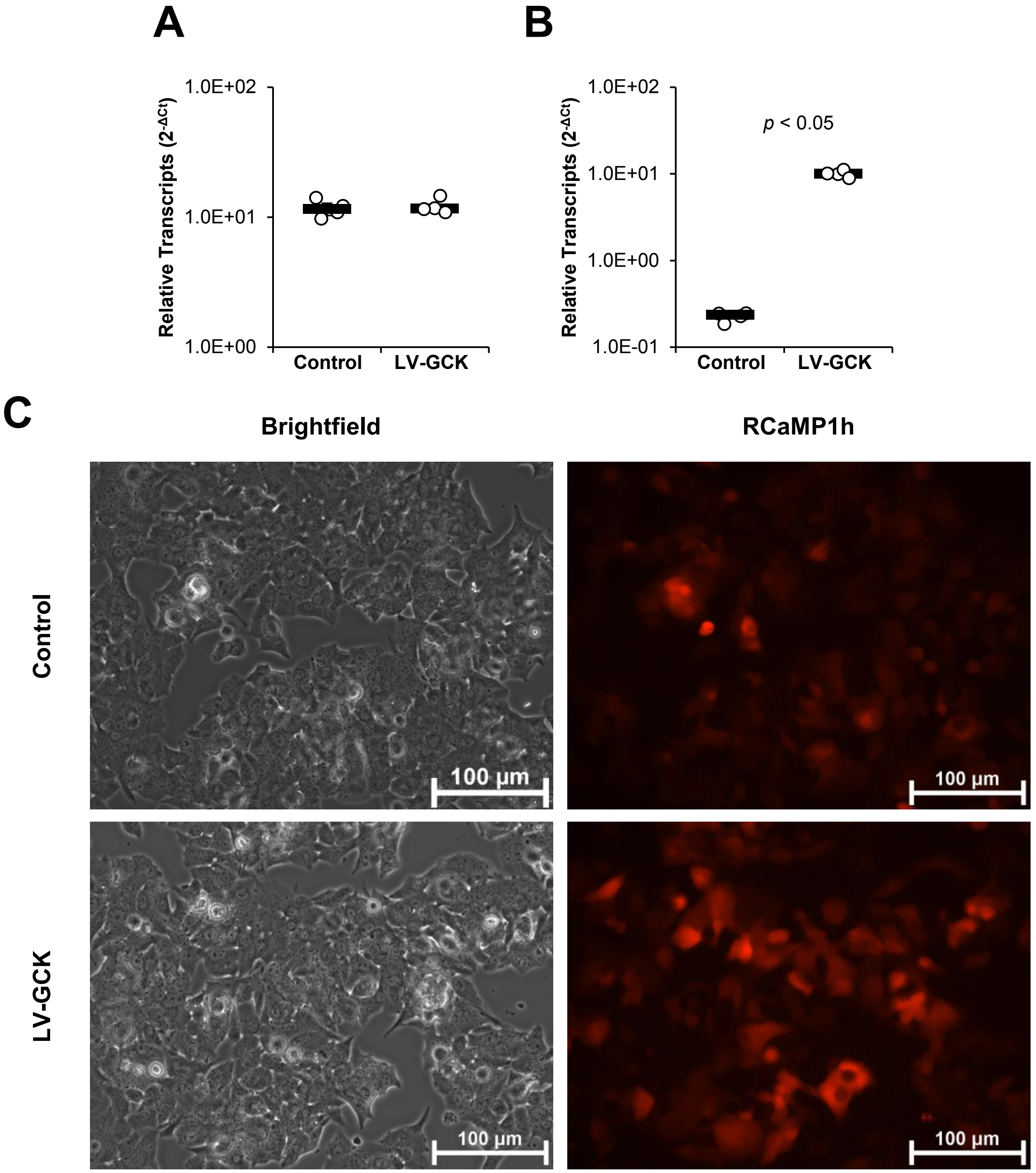

Figure S2. Min6 RCaMP1h cell transcripts and representative fluorescent images of non-transduced control and SIN-mIP2-GCK transduced Min6 RCaMP1h cells. (A) Relative transcripts of *Rcamp1h* in Min6 RCaMP1h non-transduced control cells Min6 RCaMP1h cells transduced with SIN-mIP2-GCK (n = 4 per group). (B) Relative transcripts of *Gck* in Min6 RCaMP1h non-transduced control cells Min6 RCaMP1h cells transduced with SIN-mIP2-GCK (n = 4 per group). (C) Representative fluorescent images of non-transduced control and SIN-mIP2-GCK transduced Min6 RCaMP1h cells during single cell calcium trace shown in Fig. 1D. Cells were cultured in DMEM supplemented with 10% FBS, 50 U/ml penicillin, and 50 µg/ml streptomycin. Images were taken at 10-second intervals for 20 minutes. Scale bar represents 100 µm. Results shown as scatterplot with medians (black bar) ± MAD, *p<0.05.

Disease Models & Mechanisms • Supplementary information

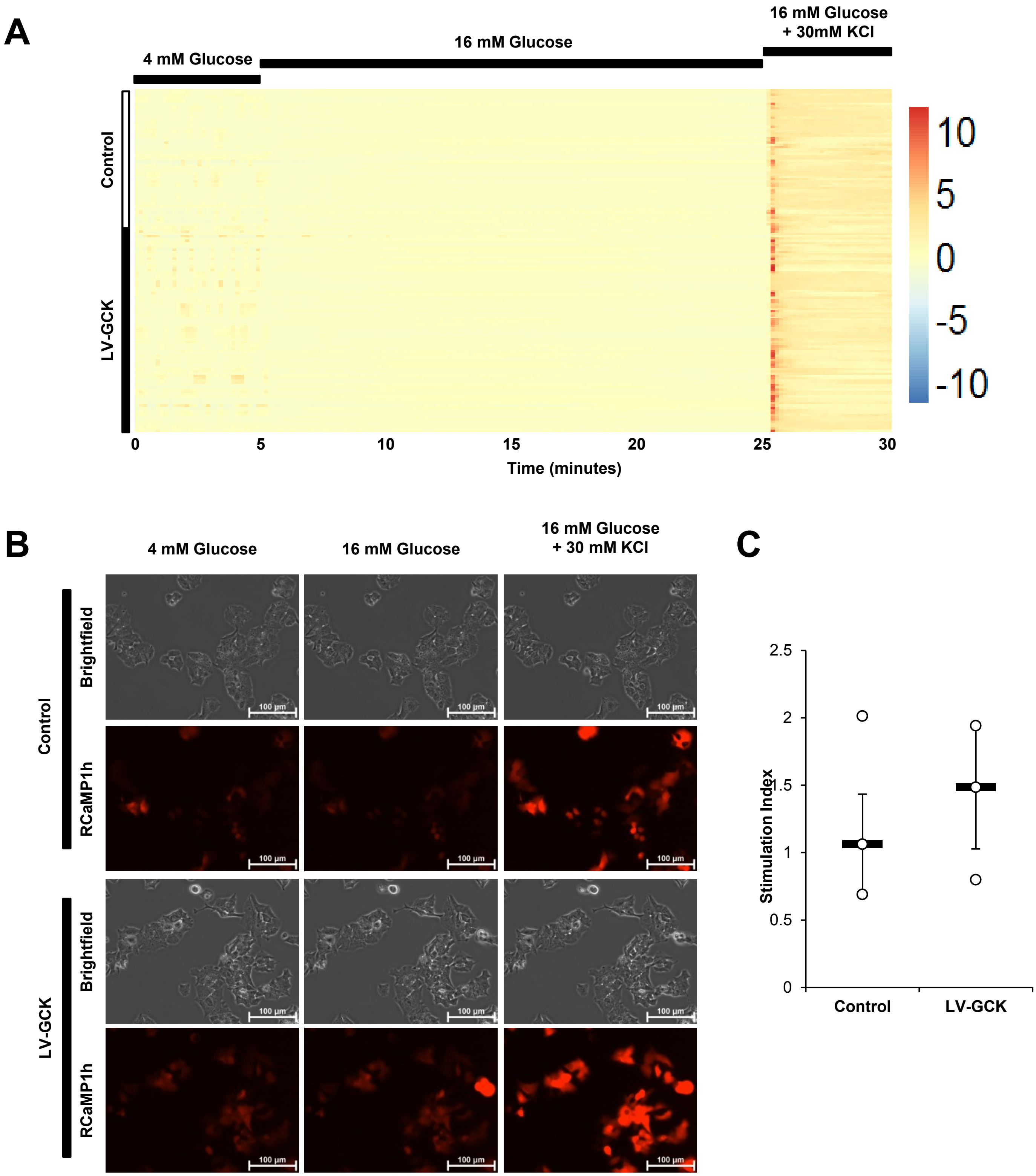

Figure S3. Single cell calcium trace and insulin stimulation index in control and SIN-mIP2-GCK transduced Min6 RCaMP1h cells. (A) Single cell RCaMP1h fluorescent intensity trace over time (n = 60-92 per group). Color scale indicates z-scores normalized per cell. (B) Representative brightfield and fluorescent images during calcium trace show in (A). Scale bar represents 100 µm. (C) Stimulation indices of non-transduced Min6 and SIN-mIP2-GCK transduced Min6 cells were measured at 4 mM and 16 mM glucose (n = 3). Results shown as heatmap or scatterplot with medians (black bar) ± MAD, *p<0.05.

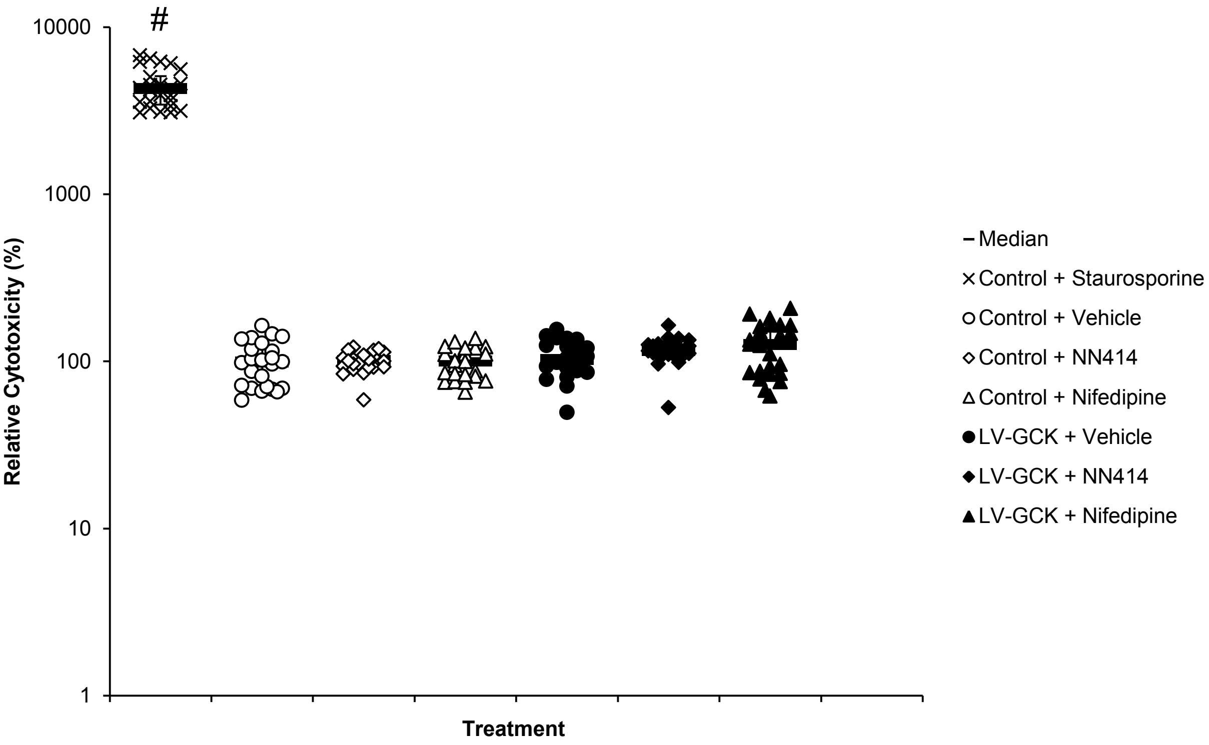

Figure S4. Cytotoxicity of NN414 and nifedipine. Non-transduced control Min6 cells and Min6 cells overexpressing GCK (LV-GCK) were incubated for 2 days with 2 µM staurosporine, vehicle, 200 µM NN414, or 28.9 µM nifedipine (*n* = 24 per group). Cytotoxocity was then assessed using the CytoTox-Glo Cytotoxicity Assay (Promega). Relative cytotoxity was calculated as luminescence prior to total cell lysis over luminescence after total cell with digitonin normalized to the respective control cells treated with the same inhibitor. Relative cytotoxicity with staurosporine was normalized to control cells treated with vehicle. Results shown as scatterplot with medians (black bar) ± MAD, #$p<0.05$ against all other groups.

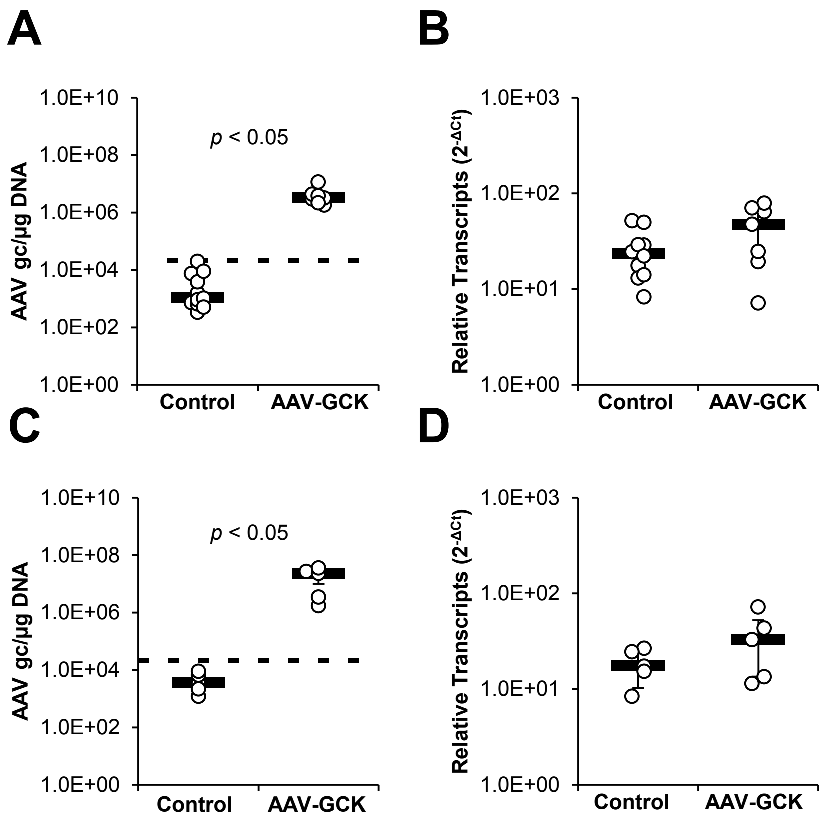

Figure S5. AAV genome copies and relative *Gck* transcripts. Mice were given PBS control or AAV-mIP2-GCK via intraperitoneal injections. DNA or RNA was extracted from pancreas sections and analyzed with qPCR or RT-qPCR, respectively, 2 weeks after injection. **(A)** AAV genome copies in mice fed chow diet (n = 7-11 per group). **(B)** Relative *Gck* transcripts in mice fed chow diet (n = 7-10 per group). **(C)** AAV genome copies in mice fed HFD (n = 5 per group). **(D)** Relative *Gck* transcripts in mice fed HFD (n = 5 per group). Dashed lines indicate limit of detection. Results shown as scatterplot with medians (black bar) ± MAD.

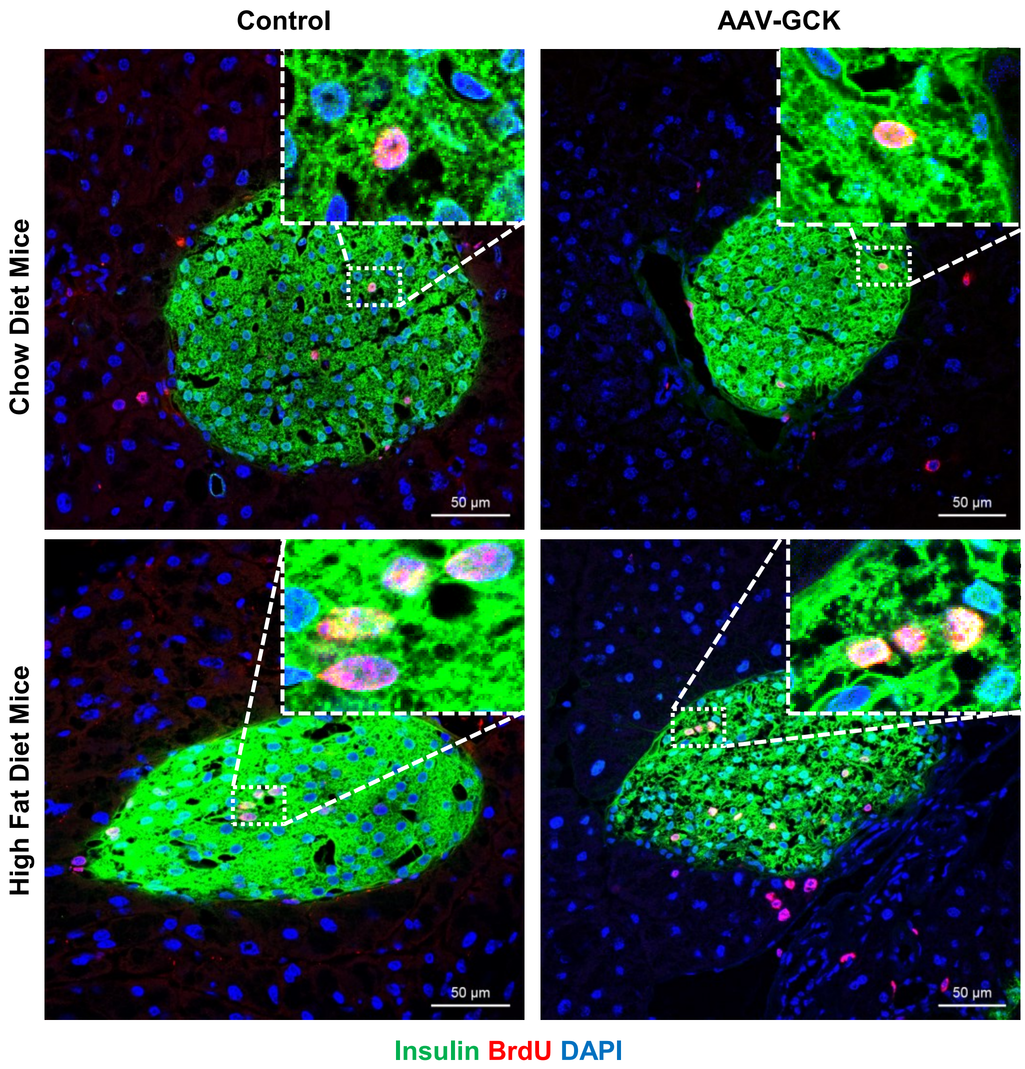

Figure S6. Representative fluorescent confocal microscopy images of pancreatic islets from PBS control and AAV-mIP2-GCK treated mice 2 weeks after injection. Proliferating β-cells were counted as insulin-positive cells co-localized with BrdU and DAPI staining. Green – insulin, red – BrdU, blue – DAPI. Scale bar represents 50 μm.

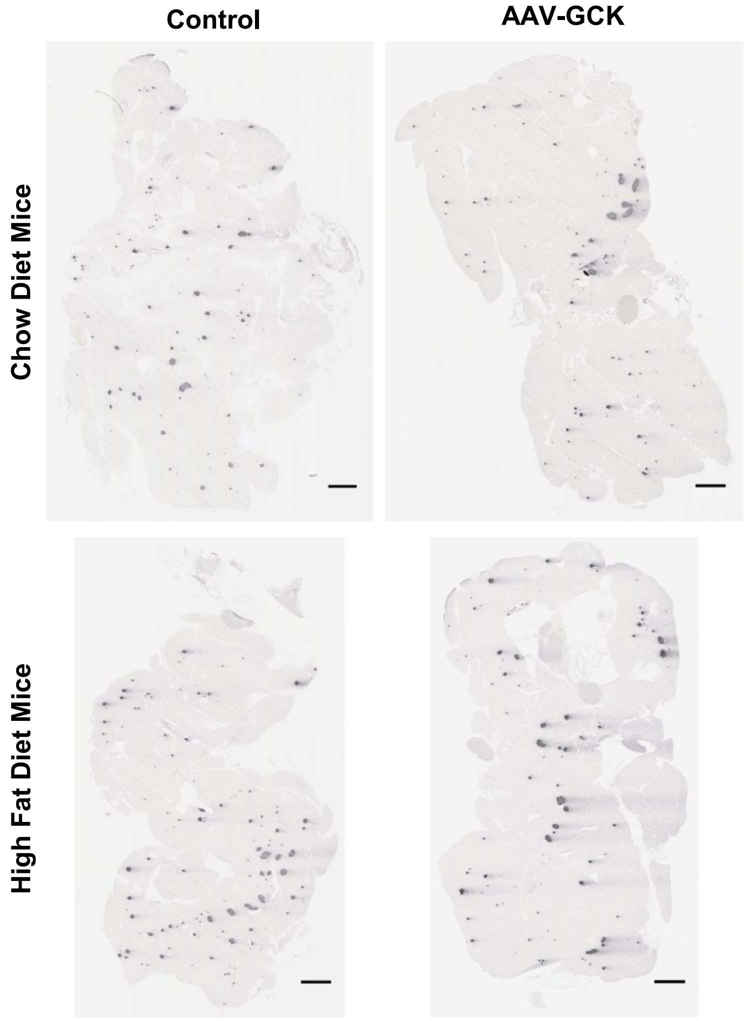

Figure S7. Representative HRP slide scan images of pancreas sections from PBS control and AAV-mIP2-GCK treated mice 2 weeks after injection. Insulin-positive area was visualized via HRP staining. Whole slides were scanned using the Aperio ScanScope AT Turbo Slide Scanner. β-cell area was quantified in Image J from scanned slide images. β-cell area was determined as the percentage of insulin-positive area as visualized by HRP staining in the entire pancreas section. Scale bar represents 1 mm.

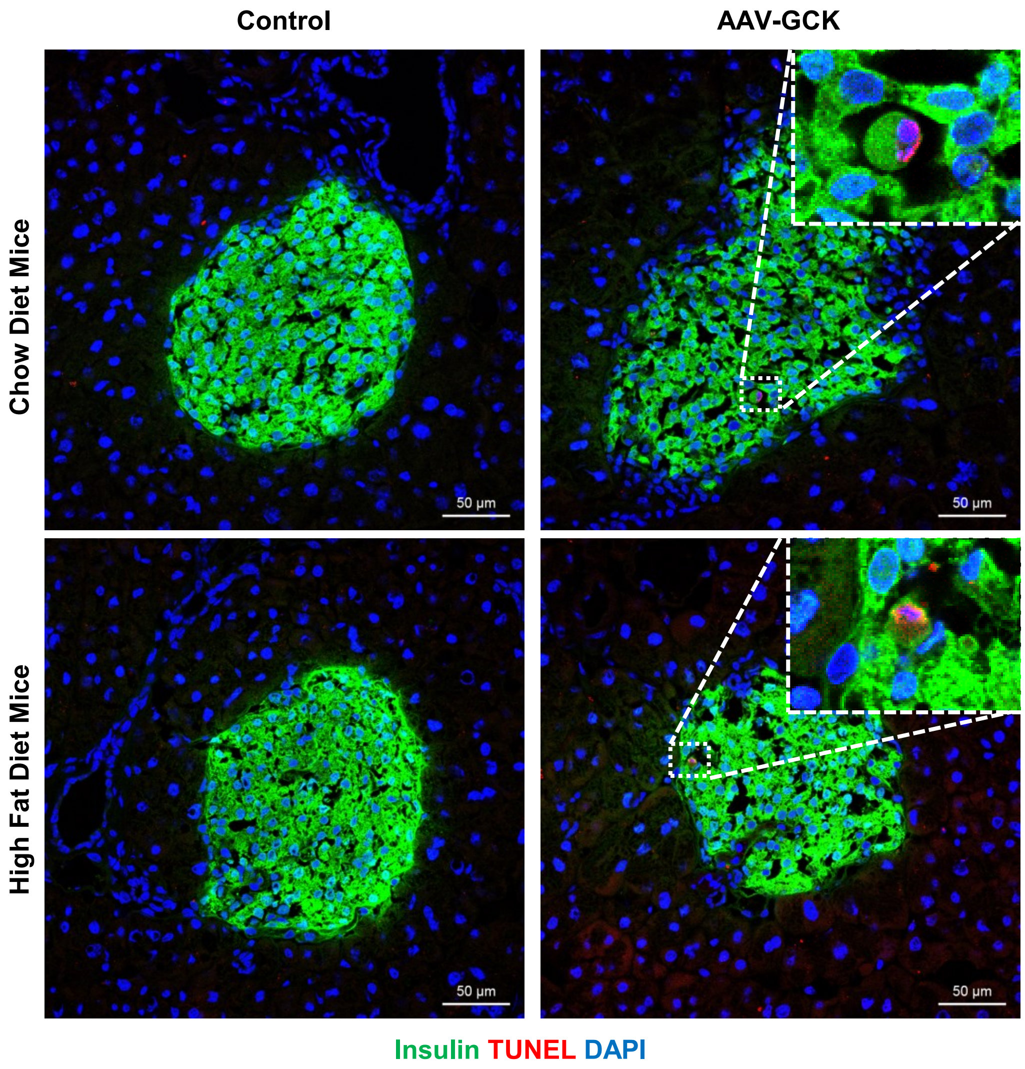

Figure S8. Representative fluorescent confocal microscopy images of pancreatic islets from PBS control and AAV-mIP2-GCK treated mice 2 weeks after injection. Apoptosis was determined using the TMR Red *In Situ* Cell Death Detection Kit (Roche). Apoptotic β-cells were counted as insulin-positive cells co-localized with Tdt-mediated dUTP-X nick end labeling (TUNEL) and DAPI staining. No TUNEL-positive cells were observed in chow diet or high fat dice control mice. Green – insulin, red – TUNEL, blue – DAPI. Scale bar represents 50 µm.

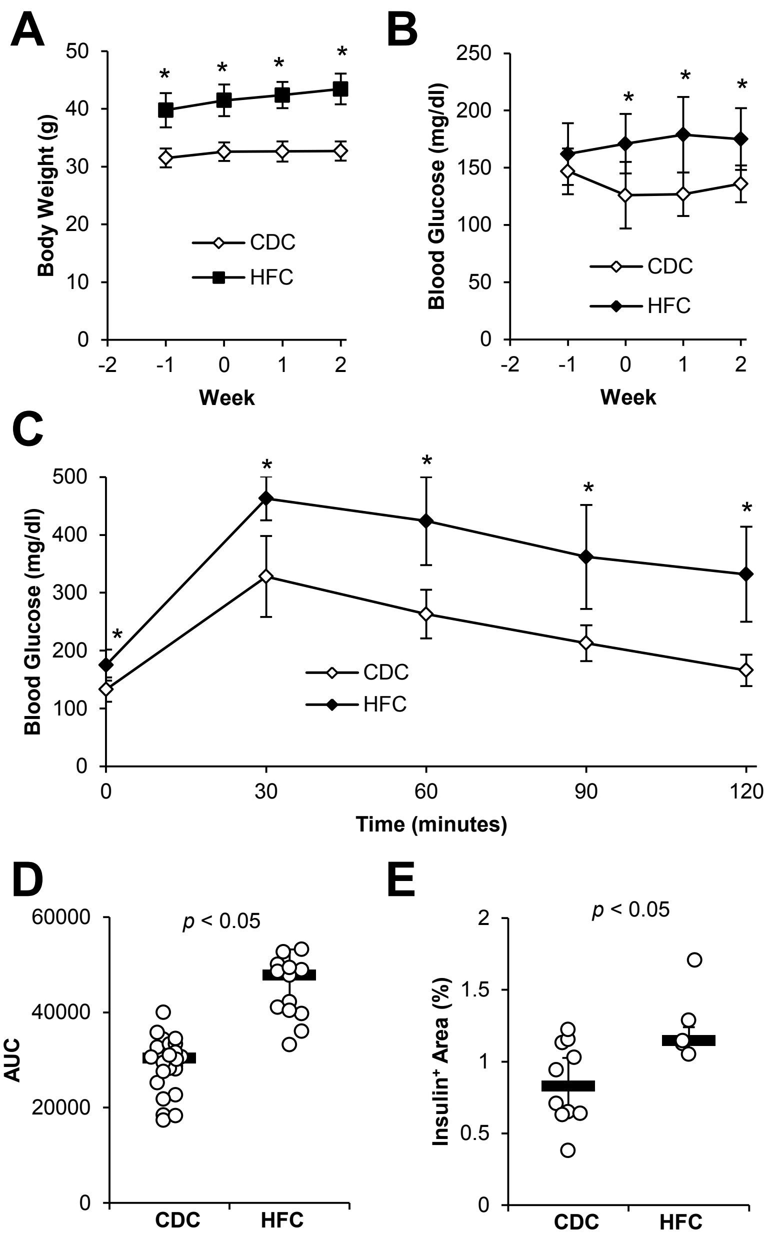

Figure S9. High fat diet feeding increases body weight and impairs glucose metabolism in mice. High fat diet was fed *ad libitum* to 6.5-month old male C57BL/6j mice for 6 weeks. Week 0 refers to the week of PBS control injection. **(A)** Weekly body weight measurements for weeks -1 (5th week of high fat diet) through week 2 (8th week of high fat diet) (*n* = 13-25 per group). **(B)** Weekly blood glucose measured after a 6-hour fast for weeks -1 (5th week of high fat diet) through week 2 (8th week of high fat diet) (*n* = 13-25 per group). **(C)** Intraperitoneal glucose tolerance test (IPGTT) after a 6-hour fast at week 2 (8th week of high fat diet) (*n* = 13-25 per group). **(D)** Area under the curve analysis for IPGTT shown in **(C)** (*n* = 13-25 per group). **(E)** Insulin$^+$ area in pancreas sections at week 2 (8th week of high fat diet) (*n* = 5-10 per group). CDC – chow diet controls, HFC – high fat controls. Results shown as line graphs median ± MAD or scatterplots with median (black bars) ± MAD, $*p < 0.05$.

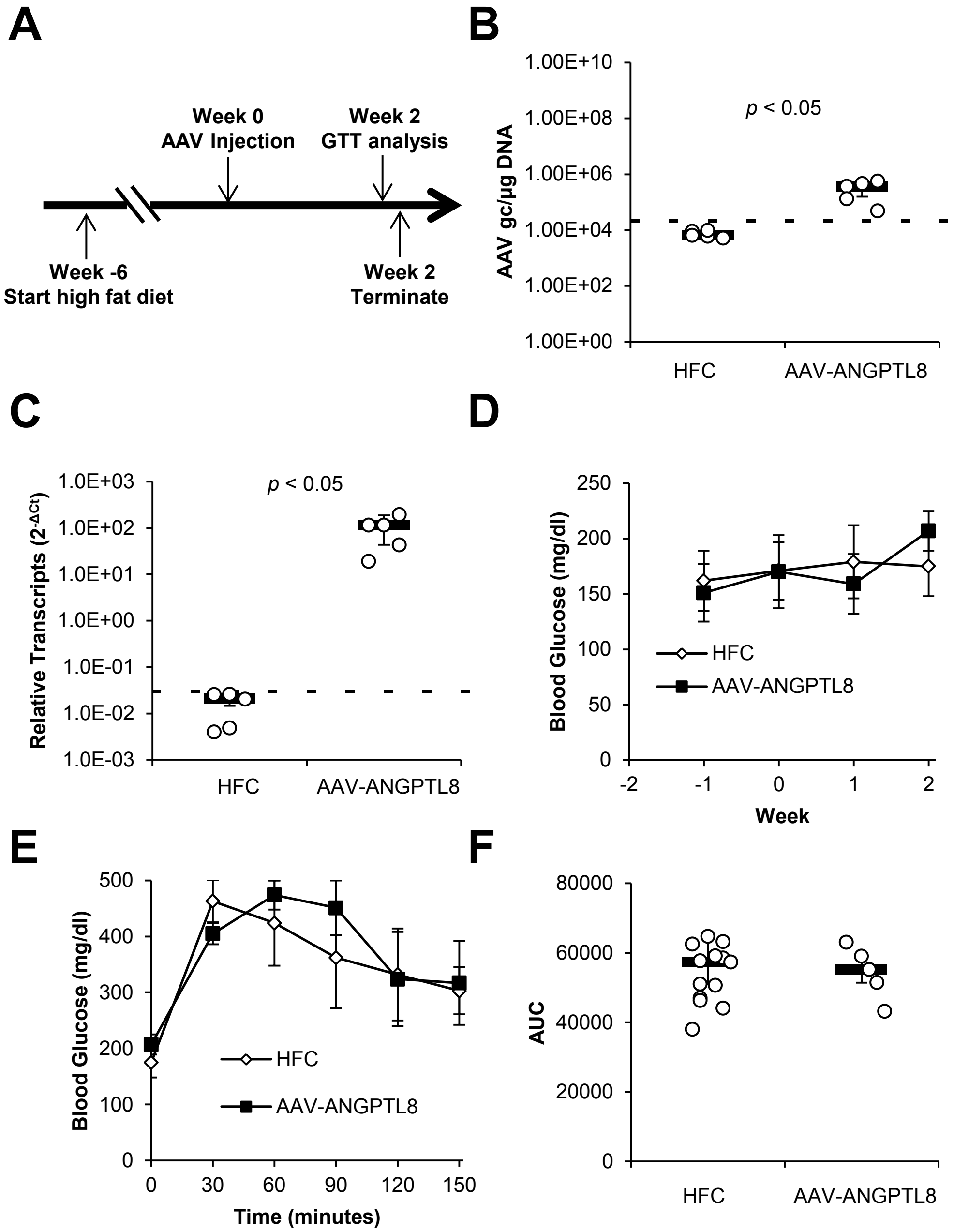

Figure S10. AAV-mediated expression of ANGPTL8 (betatrophin) does not affect IPGTT in high fat diet mice. **(A)** 6.5-month-old male C56BL/6j mice were fed high fat diet (HFD) *ad libitum* for at least 6 weeks prior to AAV injection. Mice were given PBS control or AAV-CMV-ANGPTL8 at week 0. Ubiquitous expression of ANGPTL8 in all transduced tissue was mediated by the cytomegalovirus (CMV) promoter. **(B)** AAV genome copies in pancreas (n = 5 per group). DNA was extracted from pancreas sections and analyzed with qPCR 2 weeks after injection. **(C)** Relative AAV-derived *Angptl8* transcript expression (n = 5 per group). RNA was extracted from pancreas sections and analyzed with RT-qPCR 2 weeks after injection. **(D**) Weekly blood glucose measured after a 6-hour fast (n = 5-13 per group). **(E)** Intraperitoneal glucose tolerance test (IPGTT) after a 6-hour fast at week 2 (n = 5-13 per group). **(F)** Area under the curve analysis for IPGTT shown in **(E)** (n = 5-13 per group). HFC – high fat controls. AAV-ANGPTL8 – high fat diet mice treated with AAV-CMV-ANGPTL8. Results shown as line graphs with median ± MAD or scatterplots with median (black bars) ± MAD, *p < 0.05.

Disease Models & Mechanisms • Supplementary information

Table S4. Downregulated KEGG pathways in HFD islets following GCK overexpression.

KEGG Pathway	p-value	q-value
mmu03010 Ribosome	9.40E-27	2.11E-24
mmu00190 Oxidative phosphorylation	1.68E-08	1.88E-06
mmu04510 Focal adhesion	5.36E-07	4.00E-05
mmu04110 Cell cycle	1.05E-06	5.87E-05
mmu04390 Hippo signaling pathway	4.70E-06	2.11E-04
mmu04668 TNF signaling pathway	1.54E-05	5.77E-04
mmu04115 p53 signaling pathway	2.36E-05	6.82E-04
mmu04060 Cytokine-cytokine receptor interaction	2.59E-05	6.82E-04
mmu04512 ECM-receptor interaction	2.74E-05	6.82E-04
mmu04151 PI3K-Akt signaling pathway	7.83E-05	1.75E-03
mmu03030 DNA replication	1.10E-04	2.24E-03
mmu04350 TGF-beta signaling pathway	3.72E-04	6.94E-03
mmu00240 Pyrimidine metabolism	4.04E-04	6.97E-03
mmu00480 Glutathione metabolism	4.56E-04	7.29E-03
mmu04210 Apoptosis	5.15E-04	7.69E-03
mmu04670 Leukocyte transendothelial migration	9.26E-04	1.30E-02
mmu00520 Amino sugar and nucleotide sugar metabolism	1.12E-03	1.47E-02
mmu03050 Proteasome	1.31E-03	1.64E-02
mmu04380 Osteoclast differentiation	1.82E-03	2.15E-02
mmu03420 Nucleotide excision repair	2.00E-03	2.24E-02
mmu04650 Natural killer cell mediated cytotoxicity	2.79E-03	2.98E-02
mmu04623 Cytosolic DNA-sensing pathway	3.28E-03	3.33E-02
mmu00760 Nicotinate and nicotinamide metabolism	3.47E-03	3.38E-02
mmu04215 Apoptosis - multiple species	4.37E-03	4.08E-02
mmu03013 RNA transport	6.47E-03	5.76E-02
mmu04340 Hedgehog signaling pathway	6.68E-03	5.76E-02
mmu03040 Spliceosome	7.12E-03	5.80E-02
mmu03410 Base excision repair	7.25E-03	5.80E-02
mmu04310 Wnt signaling pathway	8.30E-03	6.41E-02
mmu00230 Purine metabolism	9.63E-03	7.19E-02
mmu04218 Cellular senescence	1.06E-02	7.66E-02
mmu04540 Gap junction	1.26E-02	8.11E-02
mmu04630 Jak-STAT signaling pathway	1.29E-02	8.11E-02
mmu04810 Regulation of actin cytoskeleton	1.29E-02	8.11E-02
mmu03008 Ribosome biogenesis in eukaryotes	1.30E-02	8.11E-02
mmu00010 Glycolysis / Gluconeogenesis	1.30E-02	8.11E-02
mmu00532 Glycosaminoglycan biosynthesis - chondroitin sulfate / dermatan sulfate	1.43E-02	8.63E-02
mmu04622 RIG-I-like receptor signaling pathway	1.46E-02	8.63E-02
mmu00533 Glycosaminoglycan biosynthesis - keratan sulfate	1.59E-02	9.16E-02

RNA-sequencing analysis was performed on islets isolated from mice fed chow diet, mice fed high fat diet, and mice fed high fat diet administered with AAV-mIP2-GCK 2 weeks following AAV administration (*n* = 2-3). Pathway analysis was performed with GAGE using the KEGG database to determine the upregulated KEGG pathways in HFD islets following GCK over-expression. Significance was considered at q-value < 0.1.

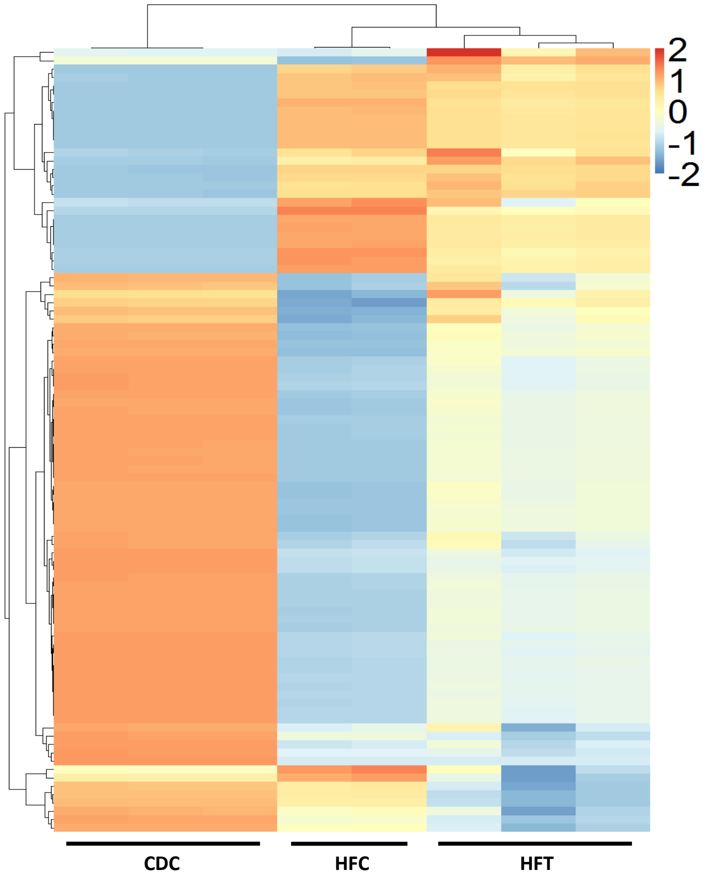

Figure S11. Heatmap of 94 genes associated with islet dysfunction (Taneera et al., 2012). RNA-sequencing was performed using RNA isolated from islets of chow diet control mice (CDC), HFD control mice (HFC), and HFD AAV-mIP2-GCK-treated mice (HFT) (*n* = 2-3 per group). Color scale shows z-scores normalized per gene.

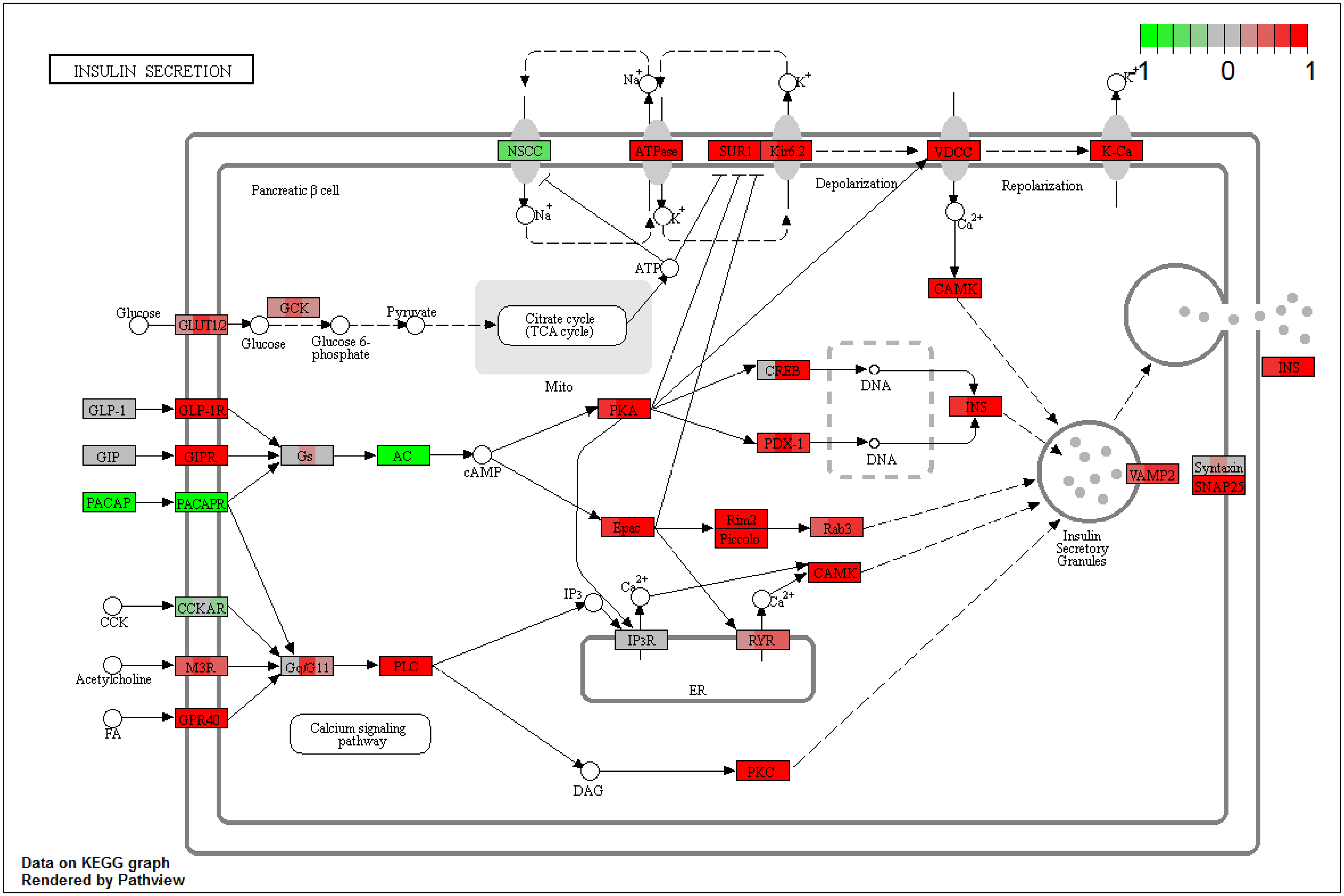

Figure S12. KEGG Insulin Secretion pathway map. Pathway analysis was performed with GAGE using the KEGG database (n = 2-3 per group). Pathway map was rendered using Pathview. Color scale represents transcript $\log_2$ fold changes of islets from HFD AAV-mIP2-GCK treated mice compared to islets from HFD control mice.

Disease Models & Mechanisms • Supplementary information

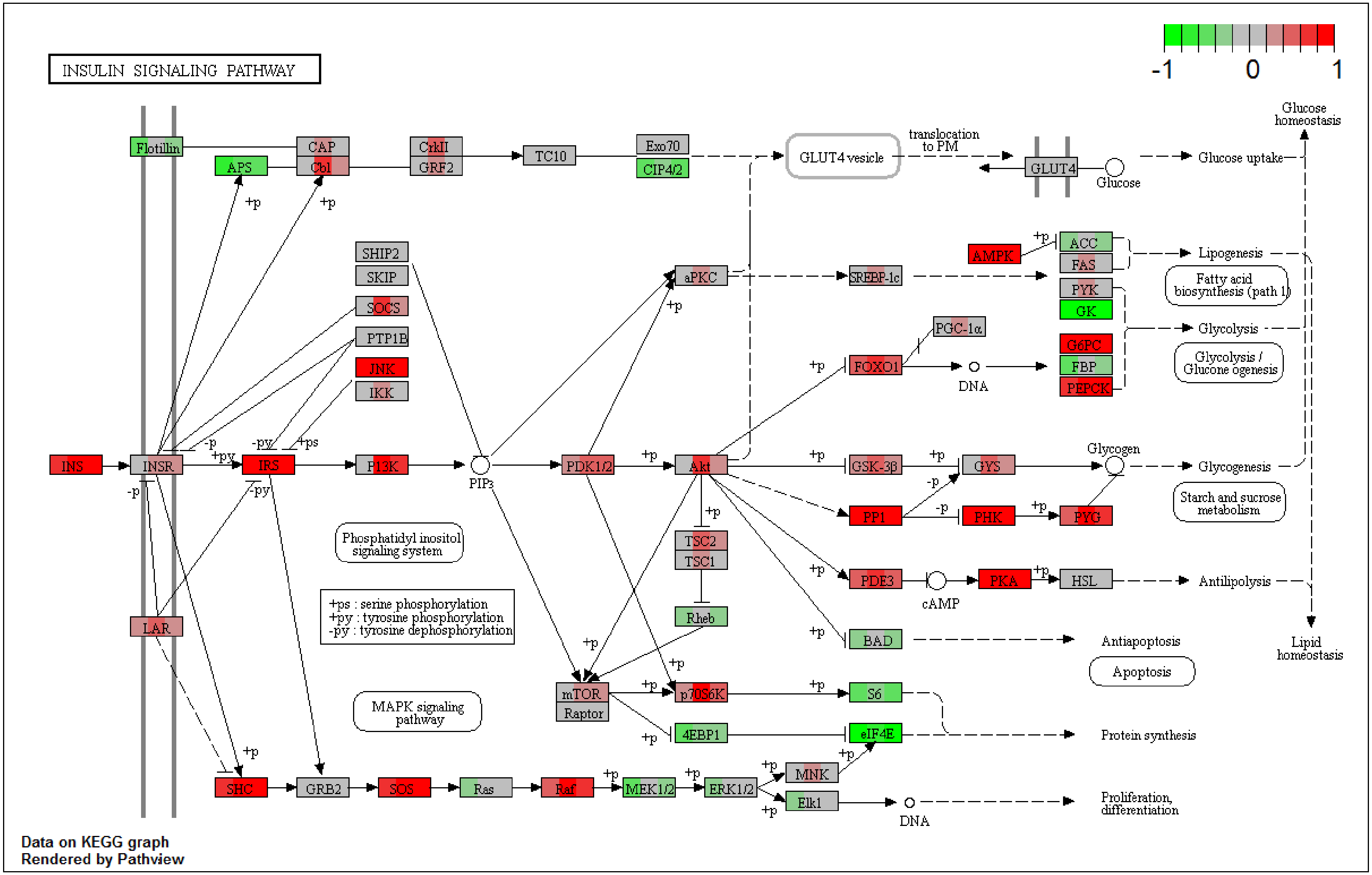

Figure S13. KEGG Insulin Signaling pathway map. Pathway analysis was performed with GAGE using the KEGG database (n = 2-3 per group). Pathway map was rendered using Pathview. Color scale represents transcript $\log_2$ fold changes of islets from HFD AAV-mIP2-GCK treated mice compared to islets from HFD control mice.

Disease Models & Mechanisms • Supplementary information

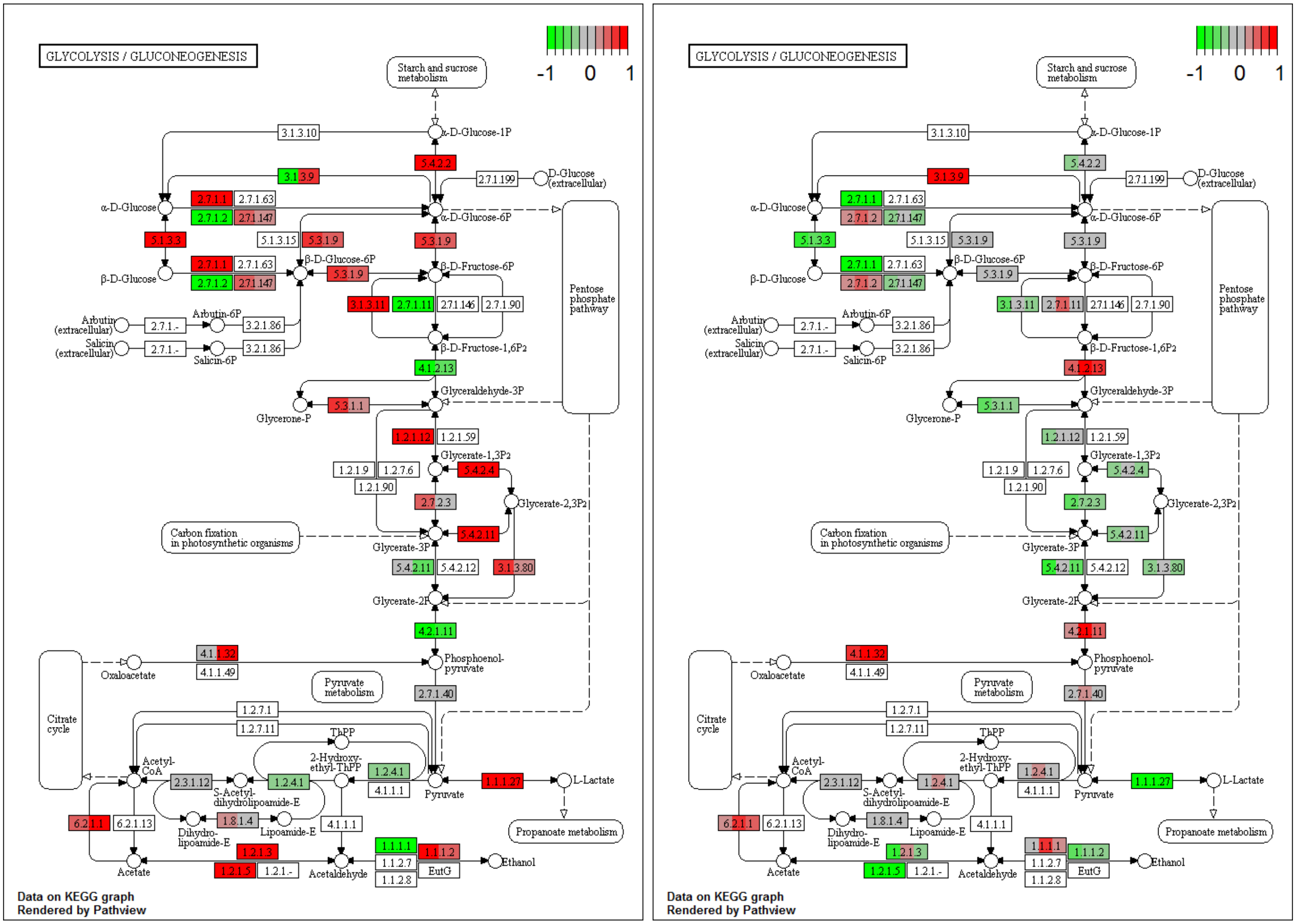

Figure S14. KEGG Glycolysis pathway map. Pathway analysis was performed with GAGE using the KEGG database (*n* = 2-3 per group). Pathway map was rendered using Pathview. Color scale represents transcript $\log_2$ fold change. Left: transcripts of islets from HFD-fed control mice compared to islets from chow-fed control mice (q-value = 6.62E-02). Right: transcripts of islets from HFD-fed AAV-mIP2-GCK treated mice compared to islets from HFD-fed control mice (q-value = 8.11E-02).

Disease Models & Mechanisms • Supplementary information

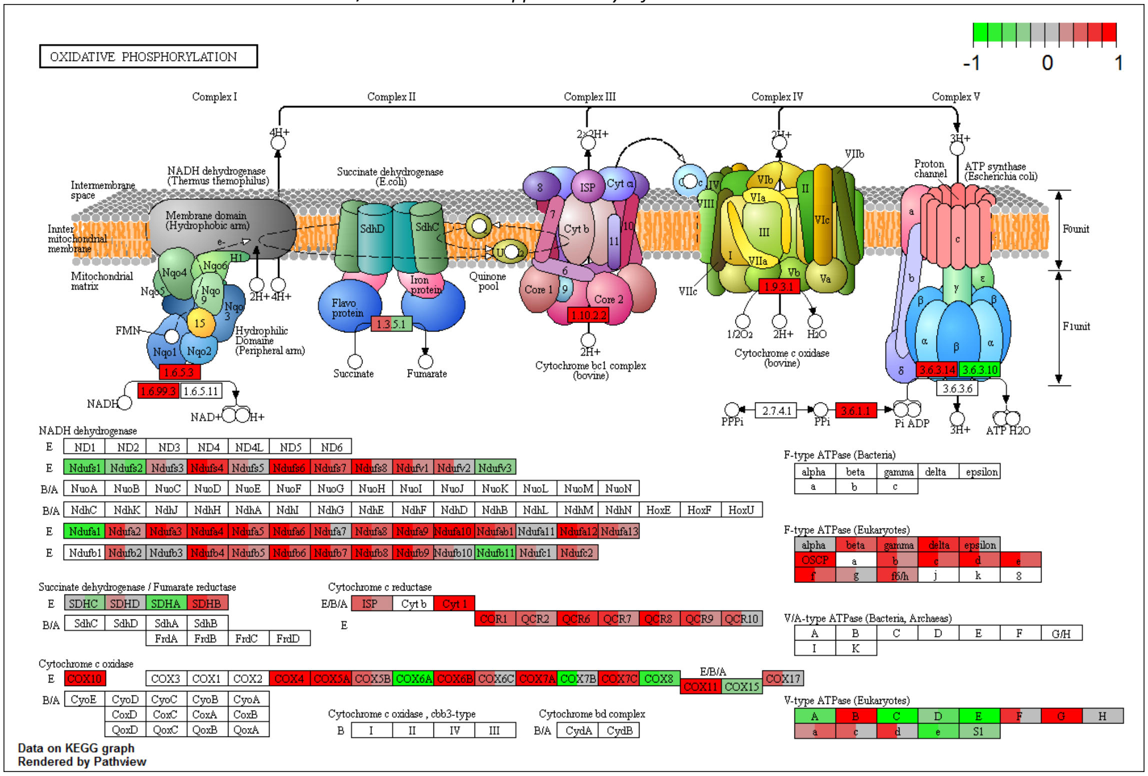

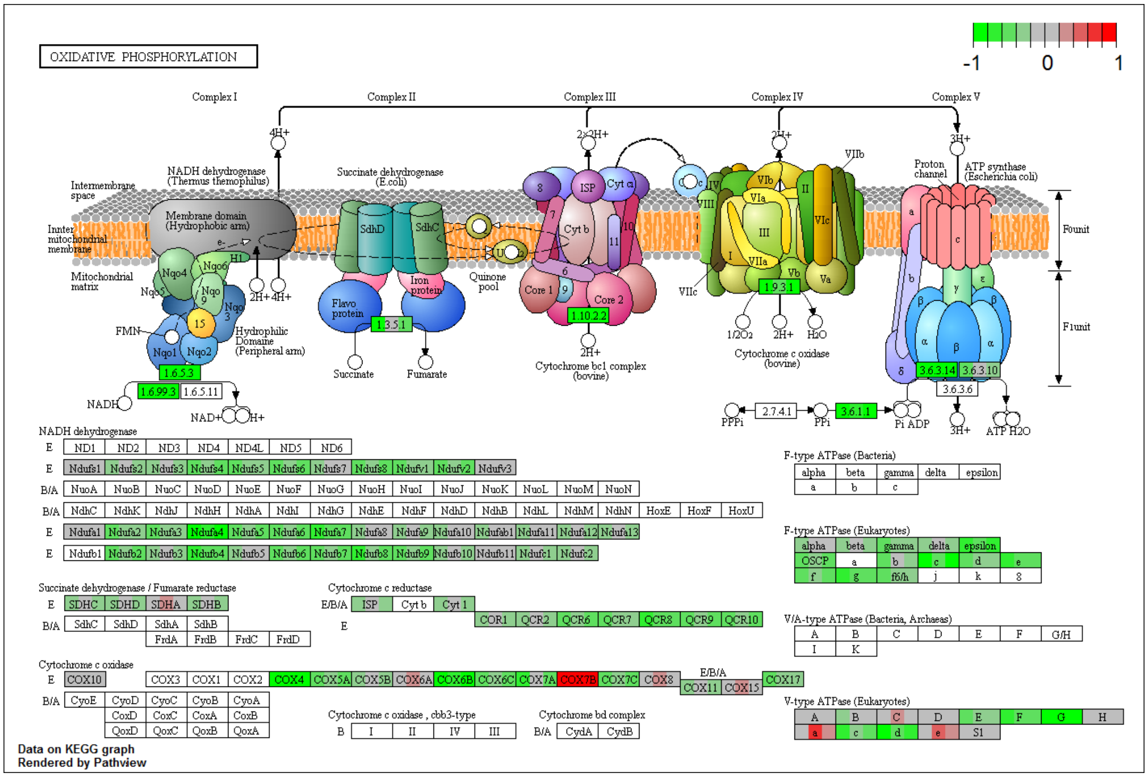

Figure S15. KEGG oxidative phosphorylation pathway map. Pathway analysis was performed with GAGE using the KEGG database (*n* = 2-3 per group). Pathway map was rendered using Pathview. Color scale represents transcript $\log_2$ fold change. Top: transcripts of islets from HFD-fed control mice compared to islets from chow-fed control mice (q-value = 8.30E-02). Bottom: transcripts of islets from HFD-fed AAV-mIP2-GCK treated mice compared to islets from HFD-fed control mice (q-value = 1.88E-06).

Disease Models & Mechanisms • Supplementary information
