# Supplementary material for: Impaired β-cell glucokinase as an underlying mechanism in diet-induced diabetes
Source: Dis Model Mech. 2018 Jun 13;11(6):dmm033316. doi: 10.1242/dmm.033316 (PMC6031355; doi:10.1242/dmm.033316)
Supplement: First Person interview [file dmm-11-033316-s2.pdf]

FIRST PERSON

First person – Brian Lu

First Person is a series of interviews with the first authors of a selection of papers published in Disease Models & Mechanisms, helping early-career researchers promote themselves alongside their papers. Brian Lu is first author on 'Impaired β-cell glucokinase as an underlying mechanism in diet-induced diabetic mice', published in DMM. Brian is a PhD student in the lab of Yasuhiro Ikeda at the Mayo Clinic, MN, USA, investigating mechanisms of β-cell function and their failures in type 2 diabetes.

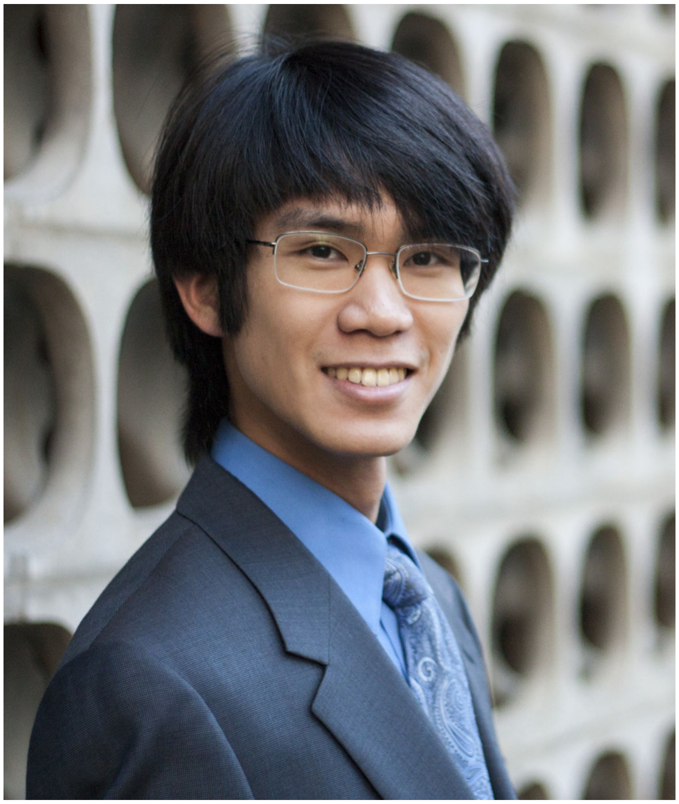

Brian Lu. Photo credit: Gary Linkevich.

How would you explain the main findings of your paper to non-scientific family and friends?

When blood sugar (glucose) is high, cells within the pancreas called β-cells secrete insulin to lower the levels of blood glucose. Glucokinase is the enzyme in β-cells that senses glucose and controls how much insulin is secreted in response to changes in blood glucose. In patients with type 2 diabetes, β-cells produce less glucokinase than normal. However, the contribution of lowered glucokinase production in β-cells to diabetes development is poorly understood. We showed that a high-fat diet reduced glucokinase production and insulin secretion in β-cells in mice. Intriguingly, when we increased glucokinase production specifically in the β-cells of high-fat-diet mice by gene therapy, we were able to restore insulin secretion. Our study therefore suggests that lowered glucokinase production in the β-cells contributes to diabetes development, and that a gene therapy approach to increase glucokinase production in β-cells may provide a novel therapy for diet-induced diabetes.

What are the potential implications of these results for your field of research?

Our research suggests that impaired β-cell glucokinase expression has an etiological role in diabetes. Our research also has potential implications for the development of glucokinase activators. As systemic pharmacological activation of glucokinase can lead to hypertriglyceridemia, more recent development of glucokinase activators has focused on organ-specific glucokinase activation. Our research showed that enhanced β-cell glucokinase expression, even without modifying glucokinase's affinity for glucose, improved the functionality of β-cells and insulin secretion. However, we also found that enhanced β-cell glucokinase expression can still result in hypertriglyceridemia. Thus, it remains possible that β-cell-specific pharmacological activation of glucokinase may also be complicated by hypertriglyceridemia.

What are the main advantages and drawbacks of the model system you have used as it relates to the disease you are investigating?

As type 2 diabetes is induced through environmental rather than genetic manipulation, induction of diabetes in mice by high-fat diet closely mimics type 2 diabetes development in humans. High-fat diet feeding induces weight gain, insulin resistance and impaired glucose tolerance within weeks in these mice. However, as diabetes development in this model depends on high-fat diet intake, the severity of diabetes in this model can vary from mouse to mouse due to differences in individual food intake. Mice also have more robust compensatory β-cell proliferation in response to high-fat diet compared to humans, which can lead to a less pronounced diabetic phenotype. Additionally, female mice are more resistant to high-fat-diet induced diabetes. Thus, we were only able to study the impact of glucokinase expression on glucose handling in males.

What has surprised you the most while conducting your research?

As glucokinase overexpression enhances proliferation without toxicity *in vitro* and has previously been shown to have a protective effect in β-cells *in vitro*, I was surprised to see increased TUNEL staining in addition to increased BrdU staining in our chow diet mice after β-cell-specific glucokinase expression. We speculate that the increase in TUNEL staining in chow diet mice could be a sign of increased glycolytic flux, as hyperglycolysis has previously been shown to induce β-cell apoptosis through p53 activation.

Brian Lu's contact details: Mayo Clinic Graduate School of Biomedical Sciences, Rochester, MN 55905, USA.

E-mail: Lu.Brian@mayo.edu

"The development of pancreas-on-chip systems may also allow researchers to model the whole human organ without using precious donor samples."

Describe what you think is the most significant challenge impacting your research at this time and how will this be addressed over the next 10 years?

One major challenge that remains in diabetes research is relating the findings in animal models back to humans. Human islets from donors are available for research but can be costly. I believe that advances in stem-cell-derived β-cells, and perhaps in the future, whole islets, will allow researchers greater accessibility to human samples. The development of pancreas-on-chip systems may also allow researchers to model the whole human organ without using precious donor samples.

What changes do you think could improve the professional lives of early-career scientists?

Proper mentoring is critical to early-career scientists as they move towards independence. I think improved networking opportunities to connect with more experienced scientists would be highly beneficial to early-career scientists.

What's next for you?

I am interested in the mechanisms of β-cell function and their failure in type 2 diabetes. After completing my PhD, I will be continuing my training in diabetes as a postdoctoral fellow.

Reference

Lu, B., Kurmi, K., Munoz-Gomez, M., Ambuludi, E. J. J., Tonne, J. M., Rakshit, K., Hitosugi, T., Kudva, Y. C., Matveyenko, A. V. and Ikeda, Y. (2018). Impaired β-cell glucokinase as an underlying mechanism in diet-induced diabetic mice. *Dis. Model. Mech.* **11**: dmm033316.
